# Reduction of appearance artifacts in wearable on-skin electronics

**DOI:** 10.1126/sciadv.aee6417

**Published:** 2026-07-15

**Authors:** Yijun Liu, Soutaro Ito, Takeo Kato, Liren Wang, Yicheng Zhu, Séverine De Mulatier, Hiroshi Arao, Shugo Suwazono, Hirotoshi Asano, Yuanyuan Zhou, Hinata Mitomo, Huimin Gong, Ohga Nomura, Gakuto Kagawa, Natsumi Watanabe, Mohammad H. Behfar, Yoshinori Kuroiwa, Tetsu Tatsuma, Yusuke Sugano, Hidetoshi Takahashi, Makoto Asai, Jiheong Kang, Naoji Matsuhisa

**Affiliations:** ^1^ Research Center for Advanced Science and Technology (RCAST), The University of Tokyo, Tokyo, Japan.; ^2^ Institute of Industrial Science, The University of Tokyo, Tokyo, Japan.; ^3^ Department of Electronics and Electrical Engineering, Keio University, Yokohama, Japan.; ^4^ School of Integrated Design Engineering, Graduate School of Science and Technology, Keio University, Kanagawa, Japan.; ^5^ Department of Mechanical Engineering, Faculty of Science and Technology, Keio University, Kanagawa, Japan.; ^6^ LIMMS/CNRS, Institute of Industrial Science, The University of Tokyo, Tokyo, Japan.; ^7^ Department of Human Sciences, Taisho University, Tokyo, Japan.; ^8^ Department of Neurology, National Hospital Organization Minami Kyoto Hospital, Joyo, Kyoto, Japan.; ^9^ College of Systems Engineering and Science, Shibaura Institute of Technology, Tokyo, Japan.; ^10^ Department of Computer Science, Kogakuin University, Tokyo, Japan.; ^11^ VTT Technical Research Centre of Finland, Espoo, Finland.; ^12^ Keio University Global Research Institute, Keio University, Tokyo, Japan.; ^13^ Department of Chemistry, Seoul National University, Seoul, Republic of Korea.

## Abstract

Facial electrophysiological signals are crucial to human-machine interfaces and health care monitoring. Soft and skin-conformable electrodes enabled long-term and comfortable signal monitoring. However, the appearance of the electrodes affects the wearer’s social interactions and self-identity, making daily usage difficult and leaving appearance artifacts. Here, we developed fully invisible and unperceivable on-skin electrodes free from appearance artifacts. Neither the wearer nor observers can detect the visual and tactile presence of the electrodes on the skin. The unperceivable property was confirmed with sensory experiments and physical characterizations of the film on skin. Furthermore, our invisible electrodes did not affect the psychological conditions of the wearers, which confirms the feasibility of artifact-free monitoring in daily lives. Last, we demonstrated the functionality of our electrode with successful monitoring of various facial electrophysiological signals, including electrooculogram (EOG), electromyogram (EMG), and electroencephalogram (EEG). Our fully invisible electrodes provide a promising direction in developing on-skin bioelectronics, seamlessly integrating health monitoring and human-computer interaction technologies into people’s daily lives.

## Teaser

Invisible electrodes reduce appearance artifacts for natural facial electrophysiological monitoring in daily life.

## INTRODUCTION

The face is the source of abundant information useful for human-machine interfaces and health care monitoring, such as electrooculography (EOG) (*1*), electromyography (EMG) (*2*), and electroencephalography (EEG) (*3*) signals, skin temperature (*4*), strain in skin (*5*), and chemicals in sweat (*6*), tear (*7*), or breath (*8*). Recently, mechanically soft electronic devices have achieved high skin conformability (*9*–*12*) and gas/sweat permeability (*10*). The soft devices enabled seamless human-machine interfaces for augmented reality and virtual reality (*11*, *12*), and high signal integrity (*13*) and long-term health care monitoring (*14*, *15*) with comfort of wear (*16*, *17*). However, their usage on a day-to-day basis, especially on the face, remains challenging due to aesthetic and privacy concerns.

Our face plays a central role in social communication (*18*). Visible electronics placed on the face would immediately draw attention, affecting the wearer’s social interactions as well as their privacy and self-identity, which we define as appearance artifacts. The appearance artifacts in the psychological conditions could lead to the contamination of measurements or even a reluctance to use the devices (*19*). Various strategies could decrease the devices’ impact on the appearance (*20*). The approaches include miniaturization (*21*–*23*), coloring like skin (*24*, *25*), and embedding the electrodes in daily objects such as clothing and earphones (*26*–*28*). Another strategy is to make electrodes with optically transparent conducting materials (*29*–*31*), which allows the larger skin-contacting areas and freedom of location.

However, these electrodes remained still very noticeable and can leave appearance artifacts for three reasons. First, a transparent skin electrode shows a larger light reflection than the bare skin. The surface of natural skin has uneven textures creating a soft diffuse light reflection, while transparent electrodes have smooth surfaces and light interference within the film resulting in a strong specific reflection of light (*32*). Replicating skin-like structure and color on the substrates helps reduce the visibility, but this approach limits the compatibility with individual skin tones. Second, most stretchable and transparent conductive materials show their distinct colors on the skin, for example, the blueish color of conducting polymers (*33*) and the dark color of carbon-based materials (*34*). Third, the invisibility or unperceivability is also affected by the tactile sensation of the on-skin devices (*35*). High permeability of moisture and temperature is also crucial for long-term wearability (*36*).

**Fig. 1.**
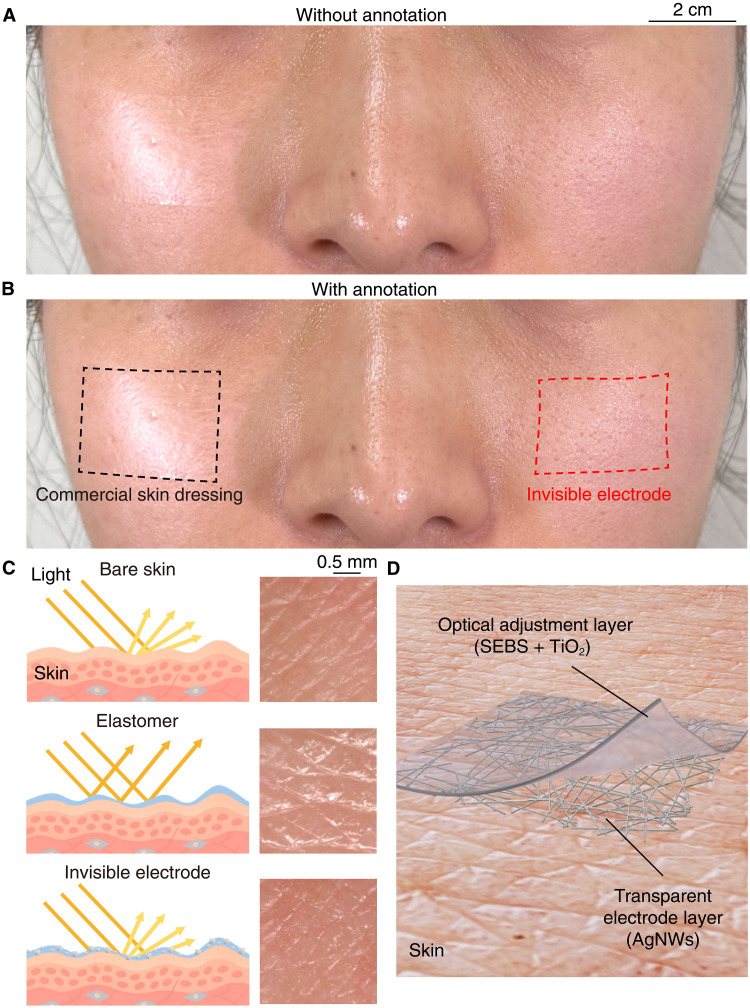
An invisible electrode. (**A** and **B**) Photo of the commercial skin dressing (left side) and the invisible electrode (right side) attached to the face without and with annotation, respectively. (**C**) Schematics (left) of incident light reflection on bare skin (top), a plane elastomer thin film on skin (middle), and on the invisible electrode on skin (bottom). The optical microscope images (right) of bare skin (top), a plane elastomer thin film (middle), and the invisible electrode (bottom). A plane elastomer thin film on the skin makes a specific reflection. The bare skin and an invisible electrode on the skin make a similar diffuse reflection. (**D**) Schematic of the invisible electrode structure.

Here, we report skin electrodes that achieve full invisibility and unperceivability to remove appearance artifacts in the psychological conditions of wearers. The electrodes are undetectable to the wearer and those around them, both visually and to the touch. Figure 1 (A and B) shows a commercially available skin film and our invisible electrode attached on each side of the volunteer’s face. The glossy texture of the commercial film makes it distinguishable on skin, whereas the limits of the invisible electrode are hardly detectable. This is achieved by the soft diffuse light reflection of our invisible electrodes, similar to that of bare skin, as illustrated in Fig. 1C. Subjective evaluations by volunteers and physical characterization confirmed that our invisible electrode has excellent invisibility, comfort, and the same tactile sensation as the skin. In addition, psychophysiological and psychological experiments verified that the invisible electrodes did not affect the psychological conditions of the wearers. Last, the electrodes on the face enable the accurate monitoring of facial electrophysiological signals, such as EOG, EMG, and EEG.

## RESULTS

### Structure of the invisible electrode

Our invisible electrode consists of two layers: an optical adjustment substrate and a transparent electrode layer (Fig. 1D). The optical adjustment substrate is an ultrathin (∼200 nm) styrene-ethylene-butylene-styrene (SEBS) film mixed with titanium dioxide (TiO_2_) nanopowders. SEBS was chosen for its low modulus and high toughness, which enables high abrasion resistance on the skin (*37*). The addition of TiO_2_ nanopowders and the ultrathin thickness (∼200 nm) allowed the optical adjustment substrate to seamlessly integrate on the skin while reducing reflections within the film, thereby enhancing its invisibility as discussed later. The transparent electrode layer is made of silver nanowires (AgNWs) whose intersections are welded to enhance the conductivity and stretchability without compromising the transparency (figs. S1 and S2) (*38*). AgNWs form mesh-like structures that exhibit high conductivity, transparency, and stretchability (*39*–*41*). We chose AgNWs due to their high conductivity and colorless transparency (fig. S3), rather than other transparent and stretchable conductors, including conducting polymers (*12*) and carbon nanotubes (*42*). Invisible electrodes were fabricated on a supporting water-soluble polyvinyl alcohol (PVA) layer and transferred onto the skin by dissolving the PVA layer (movie S1). The adhesion to the skin is comparable with previously reported skin electrodes (fig. S4) (*10*) and strong enough against weak rubbing (fig. S5). The invisible electrodes can be easily removed by scrubbing with water, which is part of a daily face wash and sufficient for 1 day of usage.

### Optical and electrical characterization of the invisible electrode

**Fig. 2.**
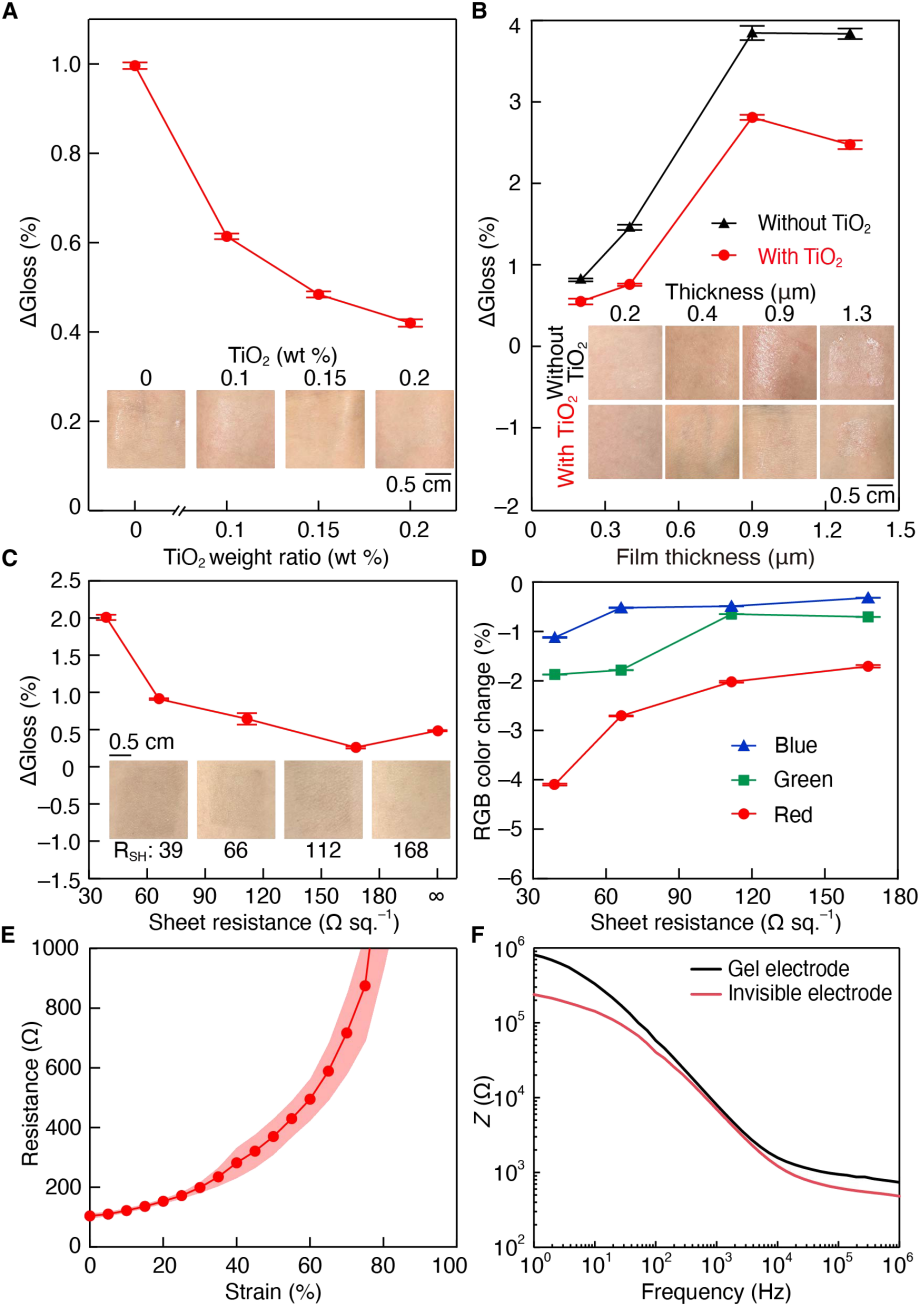
Optical and electrical properties of the invisible electrode. (**A**) Effect of TiO_2_ weight ratios in the optical adjustment substrate on the gloss values. Films with different TiO_2_ weight ratios were attached to the skin, and the gloss variation with bare skin was plotted. The thickness of the films was fixed to 0.2 μm. The top inset shows the structure of the invisible electrode. The bottom inset shows photos of the films with different TiO_2_ weight ratios on the skin. (**B**) Effect of the thickness of the optical adjustment substrate on the gloss value, without and with addition of 0.15 wt % TiO_2_. The inset shows photos of the films with different thicknesses on the skin. (**C** and **D**) Effect of the density of AgNWs on the gloss value and color on skin, respectively. The AgNWs density is reflected by the sheet resistance of the electrodes. The inset shows photos of the film with different sheet resistance attached to the skin. (**E**) Electrical resistance under strain of invisible electrodes with different density of AgNWs. (**F**) Impedance spectroscopy of gel electrodes and invisible electrodes attached to the skin. Error bars in (A) to (D) and colored region in (E) denote SD.

The addition of TiO_2_ nanopowder, as well as the low overall thickness, effectively improved invisibility on skin by reducing specular light reflection, which is characterized by gloss (note S1 and fig. S6) (*32*). Figure 2A shows the effect of the TiO_2_ nanopowder concentration on the difference in gloss when compared to natural skin. The addition of TiO_2_ reduced the gloss difference, although excessive addition of TiO_2_ caused visible white layers to appear due to the aggregation of TiO_2_ (figs. S7 and S8). The optimal concentration that maintains the original skin’s optical characteristics is 0.15 wt %. At this concentration, the gloss variation is reduced from 1.0% for plain SEBS to 0.48%. TiO_2_ nanopowders decrease gloss by scattering light due to the rough surface and high refractive index compared to SEBS (note S2) (*43*). Besides, TiO_2_ nanopowders create microscopic bumps on the surface of the optical adjustment substrate, making the film surface more effective at scattering incident light and enhancing the layer’s invisibility on skin (fig. S9). The gloss reduction was achieved regardless of the size of TiO_2_ nanopowders (fig. S10). Other oxide nanopowders—including Al_2_O_3_, ZnO, and SiO_2_—also reduced the gloss (fig. S11). In our study, TiO_2_ showed the smallest variation in gloss and red, green, and blue (RGB) values from the original skin. Figure 2B shows the effect of film thickness on the gloss values. Compared to bare skin, 200-nm-thick films showed a gloss variation of only 0.55%, while 1.3-μm-thick films showed a gloss variation of 1.9%. Thinner films resulted in reduced gloss difference because of the higher conformability and ability to reproduce the surface structure of natural skin (*44*). Thin film on the skin also maintained the original deformation of the skin while thick film regulated it (figs. S12 and S13). The addition of TiO_2_ and thin film thickness also helped maintain the original tactile sensation of the skin (fig. S14).

The effect of the layer beneath the AgNWs on the overall gloss value was also investigated by varying the AgNWs density. The AgNWs density is reflected by the sheet resistance (Fig. 2C). High-density AgNWs layer (39 Ω sq.^−1^) showed the gloss variation of 2.0% compared with bare skin, although the optical adjustment substrate alone showed 0.48%. On the other hand, the low-density AgNWs layer (168 Ω sq.^−1^) showed a significantly lower gloss difference of only 0.26%. This value was smaller than that by the optical adjustment substrate only (0.48%) on account of the increased surface roughness by AgNWs (*45*). Angle-dependent reflectance measurements further confirmed the effect of TiO_2_ and AgNWs on the invisibility (fig. S15). TiO_2_ significantly reduced reflected intensity across a wide range of detection angles compared with plain SEBS, whereas AgNWs slightly increased reflected intensity. In addition, the effect of AgNWs on the color was measured by comparing the RGB color values of an artificial skin with our invisible electrodes containing different AgNW densities (Fig. 2D). For all three RGB colors, a lower AgNW density resulted in a smaller change in color. When the sheet resistance reached 168 Ω sq.^−1^, the color change value remained below 3.3%. The sheet resistance of 168 Ω sq.^−1^ was sufficiently low compared with the skin impedance of the electrodes. Therefore, we used AgNWs with a sheet resistance of 168 Ω sq.^−1^ in the following experiments.

Figure 2E shows the resistance change of the AgNWs electrode under strain. The invisible electrodes demonstrate stretchability exceeding 70%, significantly higher than the maximum stretchability of human skin of 30% (*46*). The resistance was stable under repeated deformation of 20% strain on the artificial skin (fig. S16). The minimal variation in resistance ensures that the invisible electrodes can maintain a stable electrical signal output even during the wearer’s free movement. In addition, the skin impedance of the invisible electrode was characterized (Fig. 2F). The skin impedance of the invisible electrode was lower than that of the commercially available hydrogel electrode at 10 Hz. The impedance reduction is most obvious in the low-frequency range (10^0^ to 10^2^ Hz), which is the key frequency range for facial electrophysiological signal acquisition. The high skin conformability enables close contact with the skin texture and minimizes interface gaps (*17*, *47*).

### Sensory experiment

**Fig. 3.**
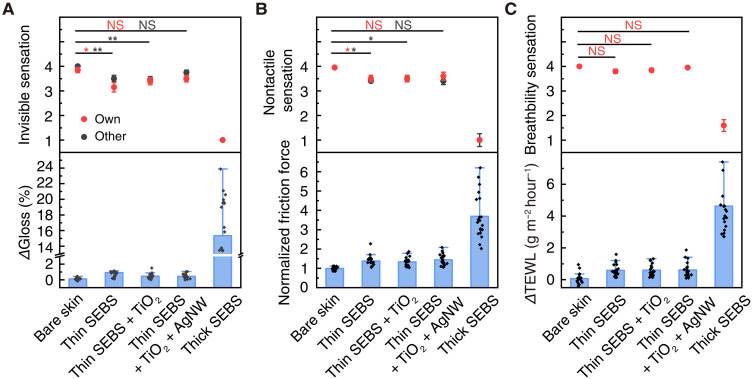
Sensory experiments to assess the unperceivability. Various thin films were attached to the skin. All the films were laminated by dissolving the PVA supporting layer. A high sensation score means high unperceivability or similarity to the bare skin. (**A**) Optical invisibility. Wearers (self) and observers (others) subjectively evaluated by sight the films attached to the wearer’s skin (top graph). Gloss variation was simultaneously measured for each film (bottom graph). (**B**) Tactile invisibility. Wearers and observers subjectively evaluated by touch the films attached to the wearer’s skin (top graph). Friction force was simultaneously measured for each film (bottom graph). In (A) and (B), thin SEBS and SEBS + TiO_2_ showed a significant difference with bare skin (*P < 0.05), whereas thin SEBS + TiO_2_ + AgNWs (invisible electrode) showed high similarity with bare skin. (**C**) Breathability. Wearers subjectively evaluated the breathability of each film (top graph). TEWL was simultaneously measured for each film (bottom graph). NS, not significant; *P < 0.05 and **P < 0.01.

The exceptional visual/tactile invisibility and the comfort of wear of our invisible electrodes were further confirmed by sensory experiments (Fig. 3). Five samples were evaluated on volunteers: bare skin, 200-nm-thick SEBS (thin SEBS), 200-nm-thick SEBS mixed with TiO_2_ (thin SEBS + TiO_2_), invisible electrode (thin SEBS + TiO_2_ + AgNW), and 100-μm-thick SEBS (thick SEBS) to explore the effects of TiO_2_ supporting film that was applied on the skin. The PVA supporting film dissolves immediately upon contact with water during transfer to the skin, and bare skin is exposed during the measurement after the same application process as the other films. The evaluation was based on both the wearer’s self sensations and others’ sensations using a scoring system ranging from one (invisible on skin) to four (obvious on skin). Self-sensation refers to evaluations by the wearers themself. Others’ sensation refers to evaluations by surrounding volunteers observing the film. Self-evaluation content included visual invisibility, tactile sensation, breathability, warmth/coldness sensation, and comfort. The gloss value, friction force, transepidermal water loss (TEWL), and epidermal temperature changes were simultaneously measured to evaluate the film’s quantitative physical properties on the skin.

Invisible electrodes had no significant difference in the invisibility and tactile sensation compared with bare skin in both self sensations and others’ sensations, with a *P* value of over 0.05 (Fig. 3, A and B). On the other hand, thin SEBS showed significant differences compared to bare skin (**P < 0.01). Thin SEBS + TiO_2_ showed a slight difference with bare skin (*P < 0.05). The sensory results for optical and tactile invisibility agreed with the measured changes in gloss and friction force, respectively. The optical invisibility of our electrodes remained consistent across the skins of volunteers with different ethnicities, genders, and ages (figs. S17 to S19). The gloss variation caused by the invisible electrode remained below 0.7% for all the volunteers. The materials and thickness of our electrodes did not require adjustment across the various skin tones and textures. The invisible electrodes also maintain high invisibility on distinct skin features, such as freckles, scars, and acne (fig. S20). Besides, the thin SEBS, thin SEBS + TiO_2_, and thin SEBS + TiO_2_ + AgNWs film showed no significant difference in breathability sensation (Fig. 3C and fig. S21), warmth/coldness sensation (figs. S22 and S23), and comfort sensation (fig. S24) because the films were very thin. Volunteers reported a stuffiness sensation from the thick SEBS film due to the reduced TEWL, which also affected the volunteers’ feeling of skin temperature. Compared to thin films, the thick SEBS does not closely fit the surface of skin, leaving an uncomfortable feeling during wear. These sensory experiments confirmed that our invisible electrode achieved complete invisibility in both visual and tactile aspects and met the requirements for long-term wear.

### Appearance artifact caused by the electrodes in the psychology of wearers

**Fig. 4.**
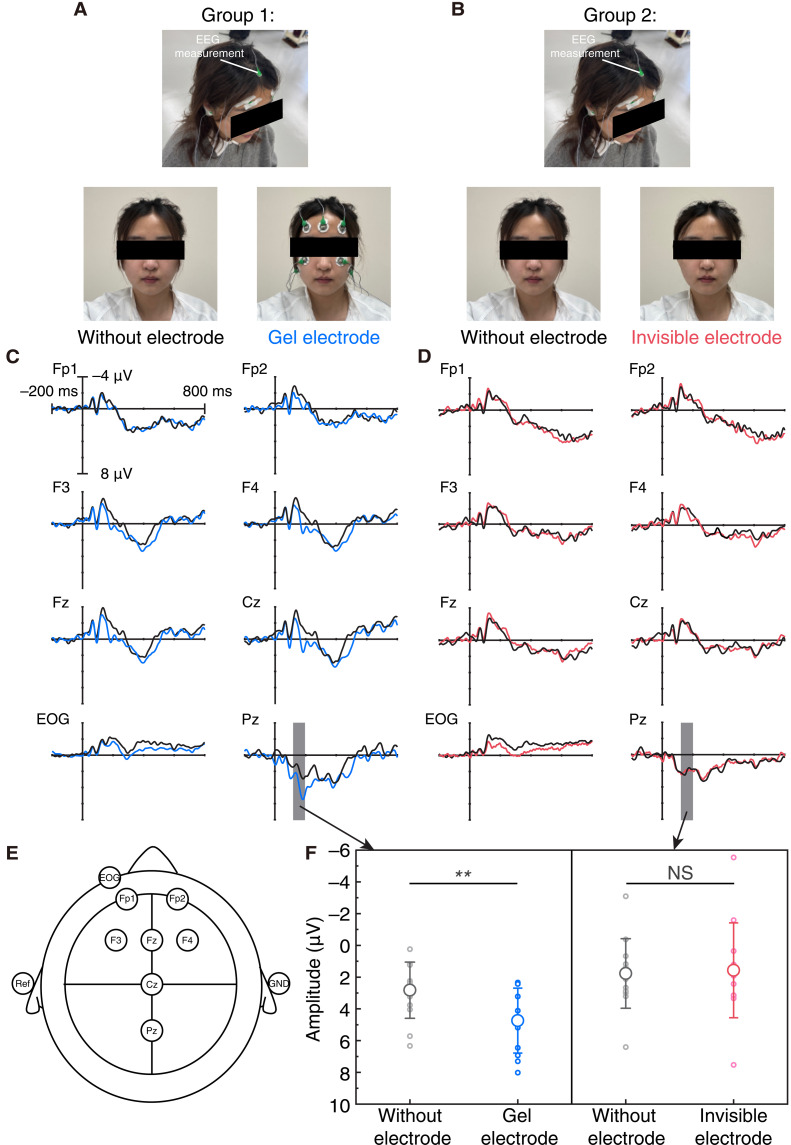
A psychophysiological experiment to assess the appearance artifact of electrodes in the psychological conditions of wearers. The experiment consisted of two sessions in a counterbalanced order between volunteers. In each session, pictures of each volunteer wearing a specific type of electrode or without any electrode were presented in random order (100 times for each condition). (**A**) Photograph of volunteers wearing active-type electrodes for EEG recording while viewing photographs of themselves with and without electrodes to examine psychological changes (top graph). Volunteers were shown two sets of pictures of herself/himself, including without electrodes and others wearing either gel electrode or invisible electrode, to decide whether acceptable or not for going outside (bottom graph). (**B**) The same task for pictures wearing the invisible electrode or without any electrode. (**C**) Grand-average ERP waveforms for the without electrode condition (black) and the gel electrode condition (blue) (*n* = 10). (**D**) Those for the without-electrode condition (black) and the invisible electrode condition (red) (*n* = 10). The grand-average ERPs were obtained by averaging the individual-mean ERPs computed from epochs without artifacts (−200 to 800 ms relative to the picture onset). (**E**) Schematic of EEG electrode locations for the psychological experiment EEG measurement. (**F**) Repeated-measures analysis of variance (ANOVA) results of the averaged waveform amplitudes at Pz (160 to 200 ms) under different electrode conditions (*n* = 10) (**P < 0.05).

With our invisible electrodes and commercial visible gel electrodes, we confirmed that the visibility affected the result of psychophysiological and psychological experiments (Fig. 4 and fig. S26), which we call appearance artifact. EEG signals, which reflect the volunteers’ psychological status, were recorded, while the volunteers were presented with two groups of facial photographs of themselves. The first group included a photo of their face without any electrodes and a photo with gel electrodes and the cables (Fig. 4A). The second group included a photo without electrodes and a photo with the invisible electrodes and the ultrathin copper wires (Fig. 4B). Volunteers were asked to imagine themselves walking through a crowded street with their face appearing as in the photo and to assess whether they would feel uncomfortable. For the EEG recording, reliable gel electrodes were placed following the 10-20 system at eight positions—front polar (Fp1 and Fp2), frontal (F3 and F4), midline frontal (Fz), central (Cz), midline parietal (Pz) (*48*), and below the left eye—for EEG and EOG signal recording (Fig. 4, A and E). The reference electrode was located at left earlobe and the ground was located at right earlobe. Another oddball task verified the stability of the event-related potential (ERP) measurement system and whether the volunteers’ cognitive responses met the standards (*49*) (fig. S25).

The visibility of facial electrodes significantly influenced the psychological conditions of wearers. The ERPs obtained when volunteers viewed a photo without electrodes and with gel electrodes showed noticeable difference in Fp2, F3, F4, Fz, Cz, and Pz (Fig. 4C). This change was especially noticeable in Cz and Pz. Furthermore, an increase in P2 amplitude was observed in the “gel electrode” set in the Fz during 160 to 200 ms. This result indicates that additional processing, possibly related to shame or embarrassment, occurred when viewing the photo with gel electrodes (*50*). Similarly, an increase of P3 amplitude was observed at the Cz within 300 to 500 ms, further supporting this finding. In contrast, the ERPs recorded when the volunteers viewed the photo without electrodes and with invisible electrodes showed no significant difference (Fig. 4D). Our invisible electrode caused no cognitive disturbance. The Pz waveforms showed the most significant difference between the conditions with and without gel electrodes. Repeated-measures analysis of variance (ANOVA) showed a significant difference in the 160- to 200-ms ERP window (**P < 0.01; Fig. 4F). In contrast, no significant difference was observed between the results with and without invisible electrodes (*P* > 0.05). The invisible electrodes did not affect wearers’ psychological conditions.

The invisible electrodes showed no appearance artifacts in psychological conditions of wearers in another psychological evaluation experiment (fig. S26). Volunteers wore electrodes (without electrodes, with gel electrodes, and with invisible electrodes) and participated in a 5-min free conversation with a randomly assigned partner (fig. S26A). The wearers recorded their emotional state before and after the conversation using the affect grid. The affect grid is a 9 × 9 matrix, which is commonly used in emotion research, and provides a subjective assessment of emotional state based on two dimensions: valence and arousal (*51*). In addition, both the wearers (Own) and partners (Other) completed a questionnaire rating how they psychologically felt during the conversation using a scale from 1 (very uncomfortable) to 10 (very comfortable) after the conversation.

Before the conversation, the emotional states of the wearers with and without any electrodes were located around the center point, representing an emotionally neutral state (fig. S26B). Nonparametric analysis (Friedman’s test) revealed no statistically significant differences among the baseline states. After the conversation (fig. S26C), both the “without electrode” group and the “invisible electrodes” group shifted toward the higher valence region, indicating a more positive emotional state, with no significant difference between them (*P* > 0.05). In contrast, the “gel electrodes” group shifted toward the lower valence region, showing a significant difference compared with both of the other groups (*P < 0.05). These results indicate that invisible electrodes caused no psychological distress during conversation. The questionnaire-based comfort evaluation further confirms this finding (fig. S26D). Both wearers (Own) and their conversation partners (Other) reported significantly lower comfort scores when the wearers worn gel electrodes compared with invisible electrodes (**P < 0.01). These psychological evaluation results demonstrate that wearing the invisible electrode has no negative impact on psychological states during social interaction, highlighting their potential for seamless integration into daily social activities without causing psychological distress.

### Facial electrophysiological signals measurement

**Fig. 5.**
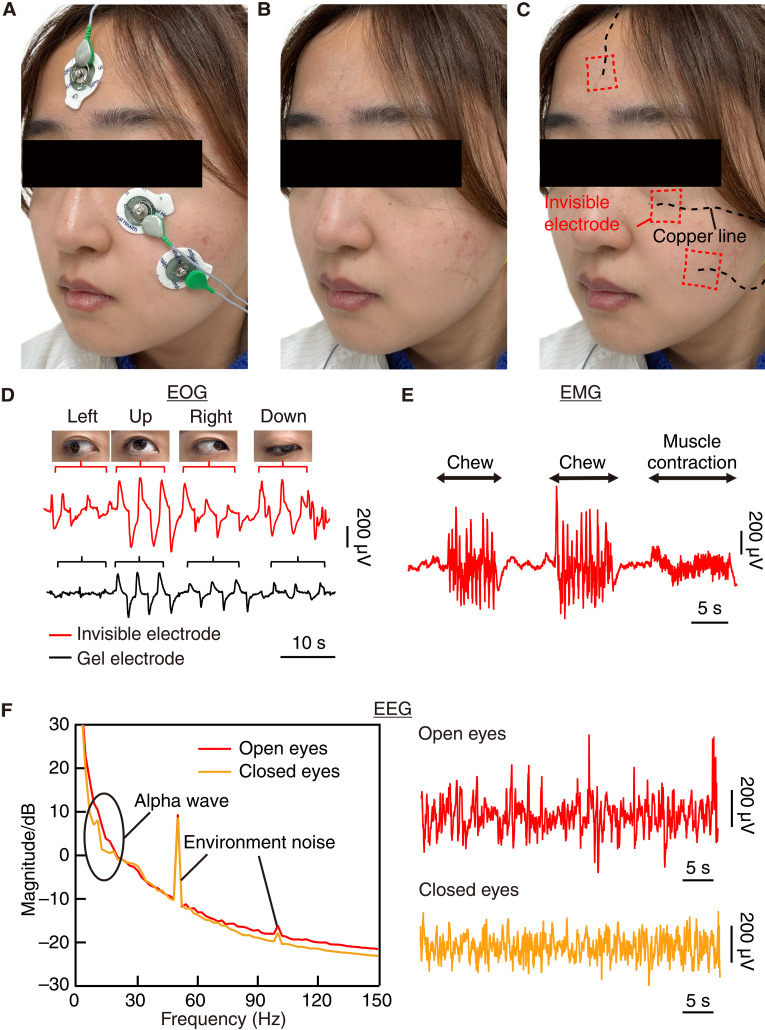
Facial electrophysiological signals monitoring by invisible electrodes. (**A**) Photo of gel electrodes attached to the face for measuring EOG, EMG, and EEG signals. (**B** and **C**) Photo of invisible electrodes placed on the face for measuring EOG, EMG, and EMG signals without and with annotation, respectively. (**D**) EOG signals were measured with an invisible electrode and a gel electrode. (**E**) EMG signals are measured with an invisible electrode. (**F**) EEG signals were measured with an invisible electrode with open and closed eyes. The left graph shows the frequency spectrum of the EEG signal. Right signals are the raw EEG signals in closed- and open-eyes states. Alpha wave fluctuations were clearly observed in the 8- to 13-Hz frequency range, and noise signals caused by external power appeared in the 50s and 100-Hz frequency bands.

We demonstrated the usability of our invisible electrodes through facial electrophysiological signal measurements (Fig. 5). Figure 5A shows commercial gel electrodes on the face for the detection of EEG, EOG, and EMG, which significantly affects the wearer’s appearance. Figure 5 (B and C) shows our invisible electrodes placed in the same positions of the face as Fig. 5A. The invisible electrodes were connected to the wireless sensing system using ultrathin copper wires, which resemble human hair and maintain the invisibility of the entire system (fig. S27). Tiny gold contact pads were introduced between the electrodes and copper wires to stabilize the electrical contact (fig. S28). The skin impedance gradually increased after repeated frictions but remained within a stable range for electrophysiological monitoring (fig. S29). The entire system enabled stable and reliable acquisition of electrophysiological signals.

EOG, EMG, and EEG signals were successfully measured with our invisible electrodes. Fig. 5D and fig. S30 show the EOG signals obtained by commercial hydrogel electrodes and our invisible electrodes during eye movements and blinking (*52*). The peak-to-peak amplitudes and signal-to-noise ratios (SNRs) of the signals recorded by the invisible electrodes were significantly larger than those of the gel electrodes (fig. S31). The averaged SNR by gel electrodes and invisible electrodes was 33 and 45 dB, respectively (*n* = 100). This benefits from the lower skin impedance of the invisible electrode in the low frequency range, enabling clear and reliable acquisition of EOG signals. EOG signals were also recorded, while the wearer performed various activities to verify the stability of signal acquisition under body motion (movies S2 and S3). The invisible electrode maintained clear signals output during walking and jumping, with only minimal fluctuation caused by motion (fig. S32). The baseline fluctuation can be reduced by miniaturizing the electronics and stabilizing the connection (*53*). Furthermore, long-term EOG monitoring was conducted for 12 hours in daily life, including research work, walking, dinner and chatting, and watching a movie (fig. S33). Both the skin impedance and optical properties of the invisible electrodes remained stable for 12 hours (figs. S34 and S35). In addition, no visible skin irritation, such as redness or inflammation, was observed during wear or after removal following 12 hours of use (fig. S36), indicating good skin compatibility for long-term use. This stability over half a day is sufficient for most of the application scenarios, as the electrodes are expected to be removed for daily face wash. We further verified the accuracy of EOG signals by simultaneously observing the movement of eyeballs with a digital camera and an infrared camera-based videooculography system (fig. S37).

Figure 5E shows EMG signals obtained with the invisible electrode attached to the cheek. The invisible electrode can be used to differentiate muscle activities such as chewing and controlled jaw clenching. The invisible electrode exhibited a higher signal amplitude than gel electrodes on account of low skin impedance (fig. S38). Last, we attached our invisible electrodes to the volunteer’s forehead to capture EEG signals in open and closed eyes conditions (Fig. 5F) (*54*). The collected EEG signals were processed using the Pwelch algorithm. The invisible electrodes effectively detected the alpha wave activity of the brain around the 8- to 13-Hz frequency range in the relaxed, closed-eyes state. The invisible electrode also exhibits slightly higher spectral magnitude in the alpha wave range (8 to 13 Hz) compared to the gel electrode (fig. S39). In addition, EMG, EOG, and EEG signals were able to be recorded simultaneously (figs. S40 and S41). The invisible electrodes demonstrated the feasibility of precise facial electrophysiological signal collection without interfering with the wearer’s daily life.

## DISCUSSION

Our study has successfully demonstrated that invisible electrodes perform well in invisibility and signal measurement. The result was compared with previously reported skin electrodes (table S1) (*35*, *39*, *52*, *55*–*61*). This work is the first report that achieves full invisibility to the best of our knowledge. Although there are some reports on on-skin highly transparent electrodes (*39*, *40*, *52*), our work uniquely addressed a skin-like gloss and tactility. This is achieved by the very high skin conformability from ultralow flexural rigidity (thinness and low Young’s Modulus) (*44*) and the addition of TiO_2_ nanopowders. While low sheet resistance was achieved in mesh gold nano-electrodes (1 Ω sq.^−1^) (*56*) and gold nanofibers (7 Ω sq.^−1^) (*57*), our value (168 Ω sq.^−1^) is low enough for electrophysiological monitoring. The lower skin resistance also ensures that the high-quality electrophysiological electrical signals can be recorded. The stretchability of the invisible electrode (70%) is comparable to that of similarly nanometal constructed mesh gold nanoelectrodes (80%) (*56*) and stretchable transparent electrodes (100%) (*39*) and surpasses the stretchability of the human skin. Note that our invisible electrodes for the first time demonstrated the invisibility through both physical and psychological characterizations, to the best of our knowledge. Furthermore, we demonstrated that the visibility of the electrodes strongly influences the psychological conditions of wearers. These appearance artifacts can disturb the acquisition of original signals in daily life settings but can be removed by the invisible electrodes. This study demonstrates its strong potential for application in wearable health monitoring devices, providing a promising technological pathway toward the next generation of unperceivable, skin-integrated electronics for daily use.

Despite the invisibility of our electrodes, there are still limitations in the current design. For example, usage beyond 1 day requires strong adhesion of the electrodes to the skin. An additional adhesive layer could provide stronger adhesion while maintaining conformability (*62*, *63*). Besides, sweat and sebum can accelerate the oxidation of the AgNWs during long-term use, reducing the electrode’s conductivity and negatively affecting performance and durability. Surface engineering strategies, such as a gold coating or core-shell passivation of AgNWs, can effectively prevent oxidation without compromising transparency (*64*, *65*). Furthermore, the current electrophysiological signal measurement system relies on ultrathin copper wires for signal transmission, which remain mechanically fragile and visually slightly noticeable. The use of ultrathin and stretchable wiring can improve the reliability and invisibility (*66*). The invisible electrode introduces a promising development direction for wearable electronics. Future research could extend the invisibility to various sensors, such as strain, temperature, and chemical sensors to monitor signals during social interactions without generating any measurement artifacts (*58*).

In summary, a totally invisible electrode has been realized by developing an optical adjustment substrate and transparent electrode layer. The invisible electrode achieved a high degree of invisibility and excellent stretchability and meets the requirements for daily wear. Invisible electrode can effectively help to reduce psychological burdens, as confirmed by the wearer’s EEG waveforms analysis and subjective evaluations. The invisible electrode can collect a variety of electrophysiological signals, including EOG, EMG, and EEG, which are widely used in health monitoring, human-computer interaction interface, and related research. Invisible electrodes are expected to replace current visible wearable devices seamlessly integrate health care and human-machine interaction technology into daily life. Users control devices simply through facial muscles and eye movements, representing a key innovation in the era of health and interaction.

## MATERIALS AND METHODS

### Materials

Glass substrates were purchased from Matsunami glass Ind. Ltd. Octadecyltrimethoxysilane (OTS) was purchased from Tokyo Chemical Industry Co. Ltd. (TCI). Trichloroethylene was purchased from Sigma-Aldrich. PVA (3-88) was obtained from Kuraray trading Co. Ltd. Hydrogenated styrenic thermoplastic elastomer (SEBS, Tuftec H1051, H1221) was supplied from Asahi Kasei Corporation. Titanium (IV) oxide [nanopowder, <100-nm particle size by Brunauer-Emmett-Teller (BET) analysis, <50 nm, x-ray diffraction, ≥99.5% trace metals basis] was purchased from Sigma-Aldrich. Titanium (IV) oxide (ST-01, ST-21, ST-31, and ST-41; x-ray diameters of 7, 20, 7, and 200 nm, respectively) was purchased from Ishihara Sangyo Kaisha Ltd. Aluminum oxide (nanopowder, particle size of ∼150 mesh, pore size of 58 Å) was purchased from Sigma-Aldrich. Zinc Oxide (nanopowder, 0.02 μm) was purchased from Fujifilm Wako Pure Chemical Corporation. Silicon dioxide (nanopowder, 5- to 20-nm particle size, 99.5% trace metals basis) was purchased from Sigma-Aldrich. Silver nitrate was purchased from Sigma-Aldrich. AgNW ink (AW030-uL, diameter: 25 to 35 nm, length: 40 to 60 μm, 1 wt % in 2-propanol) was purchased from Zhejiang KeChuang Advanced Material Co. Ltd. (China). l(+)-ascorbic acid, 2-propanol, toluene, ethanol (99.5%), ammonium hydroxide, and cyclohexanone were purchased from Fujifilm Wako Pure Chemical Corporation.

### OTS glass substrate

An OTS glass substrate was first prepared as a supporting substrate for the optical adjustment substrate. A glass substrate was cleaned using oxygen plasma (PIE Scientific LLC, Tergeo-plus tabletop plasma cleaner) with a power of 150 W and an oxygen flow of 5 standard cubic centimeters per minute (sccm) for 5 min. OTS solution in trichloroethylene [OTS/trichloroethylene = 1:1000 (v/v)] was filtered with a 0.22-μm nylon filter and spin-coated on the glass substrate at 0 rpm for 30 s and at 3000 rpm for 30 s. Then, the glass substrate was treated with NH_4_OH vapor in vacuum overnight. Last, the glass substrate was subjected to a bath sonication treatment for 10 min (Emerson Electric Co., tabletop cleaners CPX 3800) in toluene and then dried with nitrogen blow.

### Optical adjustment substrate

PVA solution (25 wt % in deionized water) was spin-coated on the OTS glass at 500 rpm for 60 s and dried at 100°C for 3 min to form a sacrificial layer. SEBS H1051 pellets were dissolved in toluene at a concentration of 20 mg ml^−1^. The TiO_2_ powders (0.15 wt % TiO_2_ powders in SEBS H1051 solution) were added to the SEBS H1051 solution and dispersed by shaking the mixture for 5 min and bath ultrasonication for 1 min. The SEBS H1051:TiO_2_ solution dispersion was spin-coated on the PVA layer at 1000 rpm for 60 s and dried at 100°C for 30 min.

### AgNW layer

The optical adjustment substrate was treated with oxygen plasma with a power of 50 W and an oxygen flow of 5 sccm for 1 min. The AgNW ink was diluted in 2-propanol [1:5 (v/v) in 2-propanol] and gently shaked to evenly disperse the AgNWs in the solution. The prepared AgNW solution was spin-coated at 1000 rpm for 60 s. The intersections between AgNWs were welded with the following process (*39*). A silver nitrate solution (1 mM in ethanol) was spin-coated on top of the AgNWs at 1000 rpm for 60 s to allow the silver ions to flow to the intersections of the AgNWs. Then, an l(+)-ascorbic acid solution (1 mM in ethanol) was dropcasted and left for 1 min on the AgNWs to reduce the silver ions at the intersection. The solution was then spin-coated at 1000 rpm for 60 s. The spin coatings of silver nitrate solution and l(+)-ascorbic acid solution onto the AgNW were repeated three times.

### Transfer of the invisible electrode to the skin

The invisible electrode was peeled off from the OTS-treated glass and adhered to the skin (AgNW layer facing the skin). A moistened wiper (Texwipe Company, LLC., TX604) was placed onto the bioelectrode to dissolve the PVA sacrificial layer. After drying at room temperature, the transfer process was completed.

### Electrical properties of AgNW layer

SEBS H1221 cyclohexane solution (80 mg ml^−1^) was dropcasted onto the OTS glass substrate. The substrate was then covered with a glass petri dish to dry the solvent overnight and form a 36-μm-thick substrate. The SEBS substrate was peeled from the OTS glass substrate and fixed onto an *x*-axis stage (Coms Corporation, PM80B-200X-HQ). The invisible electrode was transferred onto the SEBS substrate by dissolving the PVA layer. The sample was longitudinally stretched at a speed of 0.5 mm s^−1^ using the position controller (Coms Corporation, CP-700D), and the electrical conductivity was measured using the LCR meter (NF Corporation, ZM2376).

### Physical properties of film on skin

The color and gloss of the films on the skin were measured using the spectrophotometer (Konica Minolta Japan Inc., CM-26dG). The spectrophotometer was placed perpendicularly to the skin, centered on the middle of the sample with a light pressure applied on the sample. The static friction measuring machine (Trinity-La Inc., i-tester TL201) was used for tactile measurement. The tactile contactor (finger model) was placed horizontally on the surface of the thin film. Tests were performed with a weight of 50 g, a distance of 10 mm, and a speed of 10 mm s ^−1^. The TEWL measurement probe (Integral Corp., Tewameter TM HEX) was gently placed perpendicularly to the skin surface and held for 25 s to measure the effect of the sample on the transepidermal water loss from the skin surface. We used a temperature sensor (Nikkiso-therm Co. Ltd., N543R) fixed to the skin where the samples were attached using medical adhesive tape for a measurement time of 1 min to evaluate the effect of the samples on epidermal temperature.

### Film adhesion to artificial skin

A three-dimensional (3D) printed mold with a 1.8 cm–by–1.8 cm window was used to support the samples. The edges of the film were fixed to the window using strong double-sided tape, while the central region (1 cm by 1 cm) was attached to artificial skin (Beaulax Co. Ltd., BIODY Plate, P001-001) using the same procedure as on human skin. Adhesion was evaluated by measuring the normal detachment force using a tensile mechanical tester (Shimadzu, EZ-LX). The supporting window was lifted at a constant speed of 10 mm min^−1^ until complete detachment from the artificial skin. All measurements were performed at room temperature.

### Angle-dependent reflectance measurements

Angle-dependent reflectance measurements were performed using a fixed incident light source (Asahi Spectra Co. Ltd., Xenon light source, LAX-102) and a movable photodetector (Ocean Optics Inc., Photodiode array detector, HR4000GC-UV-NIR). The angular distribution of reflected light was measured using a goniophotometer (TECH-World Ltd., TPM-2500). A light beam with a power of ∼9 mW cm^−2^ was directed onto the sample surface at an incident angle of 40° with respect to the surface normal, with a distance of ∼4.5 cm from the lens tip to the sample. The reflected light intensity was recorded using a detector positioned at different detection angles (θ_d_), defined from the surface normal, ranging from 30° to 70°. A white A4 sheet of paper was used as a reference to normalize the reflectance. SEBS-based films were transferred onto artificial skin using the same transfer procedure as described above. The samples were settled on the measurement plate using double-sided tape. All films had a thickness of 200 nm.

### Compliance of thin films to skin deformation

Figure S9 shows the schematic of the experiment. A 2D array was patterned on a bare skin using a skin tanner (NURBS, Lotion 01). SEBS films with different thicknesses and scotch tape were attached to the skin on top of the patterned array. The deformation was applied either by pulling both ends of the pattern outward (tensile strain) or pushing both ends of the pattern inward (compressing strain). The pattern deformation was captured by a camera and analyzed using the sampling moiré (SM) method to extract the displacement.

### Sensory evaluation experiment

This experiment has been approved by the University of Tokyo, Research Ethics Committee (approval no. E2025ALS287), and written informed consent was obtained from the volunteers. The initial state of the volunteers’ skin was recorded, including measurements of gloss value, nontactile sensation, transepidermal water loss, and epidermal temperature. Various types of films were adhered to the volunteers’ skin, ensuring perfect adherence and avoiding wrinkles or bubbles. During the attaching process, volunteers were unaware of the specific locations and types of films attached to different areas, minimizing the influence of psychological factors on their evaluation of the films. Following this, the volunteers were asked to find the films, and a sensory experiment was conducted with volunteers evaluating the wearing sensation for each film. The volunteers used the evaluation form to record their sensations and comments. Each aspect was rated on a four-point Likert scale (1: disagree, 2: slightly disagree, 3: slightly agree, 4: agree), and the evaluations included invisibility, nontactile sensation, breathability, warmth/coldness sensation, and discomfort sensation. Last, the film was removed, and the skin surface closely observed to see whether any adverse reactions such as allergies or itching appeared.

### Psychological evaluation experiment

This experiment has been approved by the University of Tokyo, Research Ethics Committee (approval no. E2025ALS287). The psychological experiment involved three conditions: without electrode, invisible electrode, and gel electrode. The electrodes were attached to seven locations on the face, including the forehead, under the eyes, and cheeks. Two locations as the ground (GND) and reference attached to the earlobes. Photos of the volunteers’ faces were taken under each condition. A white background was used, and the camera was fixed on a tripod to ensure the same position for all photos. For each condition, volunteers were asked to communicate with a randomly selected partner for 5 min. The topic of conversation was not limited. Before and after each conversation, volunteers were asked to rate their emotional state using the affect grid. The affect grid is a 9 × 9 matrix where the horizontal axis shows the level of valence, and the vertical axis shows the level of arousal. Scores range from 1 to 9, increasing from left to right and from bottom to top. After each conversation, the volunteers were asked to fill out a questionnaire to rate how they psychologically felt during the conversation on a scale from 1 (very uncomfortable) to 10 (very comfortable). Conversation partners also need to complete the same questionnaire, rating their feelings when communicating with the volunteer in each condition.

Following a short break, volunteers started the next step of the experiment. EEG electrodes were placed according to the 10-20 system at positions Fp1, Fp2, F3, F4, Fz, Cz, and Pz. An additional electrode was placed under the eyelid for EOG recording. EEG signals were recorded using the electrophysiological signals analyzer (Miyuki Giken Co. Ltd., polymate pocket MP208). Volunteers were guided to view two sets of facial photos of themselves. In one set, they were shown photos of their face without electrode and with gel electrodes. In the other set, they were shown photos of their face “without electrode” and “with invisible electrodes.” Each set contained 100 photos per condition in random order and asked to imagine walking through a crowded street with the same appearance as in the photo. If they thought they feel comfortable going outside with that look, press the left key. If not, press the right key. EEG signals were recorded at the same time to evaluate their psychological responses. Oddball group as the supplementary group was also shown to volunteers, and the EEG signals were recorded.

### Electrophysiological signals measurement

The invisible electrode system connects the electrodes to the electrophysiological signals analyzer through an ultrathin connecting copper wire [Kyowa Harmonet Ltd., ultrafine enameled wire (two types of polyurethane copper wire) of 0.12 mm]. A highly conductive 1 mm–by–1 mm in size and 45-nm-thick gold electrode sheet was incorporated between the connecting wire and the invisible electrode to enhance the connection stability and effectively prevent noise from interfering with the signal. The gold electrode was deposited onto a 1-μm-thick SEBS H1221 film by the vacuum thermal deposition process. The SEBS H1221 film was obtained from a solution of SEBS H1221 in toluene (80 mg ml^−1^) spin-coated at 1000 rpm for 60 s. The gold electrode sheet was attached to the skin using a less viscous safety latex adhesive. The ground electrode was secured at the cervical spine using a gel electrode (Cardinal Health Inc., Albo H124 form 30 mm by 24 mm), while the reference electrode, also a gel electrode, was placed on the thin muscle area behind the ear. Invisible electrodes were used as test electrodes and placed on the eyelids, cheeks, and forehead to measure facial electrophysiological signals. An eye tracker (Gazepoint Research Inc., GP3 eye tracker) was used as a reference measurement for eye tracking. A camera (Sony Group Cor., α6400) was positioned directly in front of the volunteer to record the testing process.

## Supplementary Materials

### The PDF file includes:

Figs. S1 to S41 Notes S1 and S2 Table S1 Legends for movies S1 to S3
