# Supplementary material for: Reduction of appearance artifacts in wearable on-skin electronics
Source: Sci Adv. 2026 Jul 15;12(29):eaee6417. doi: 10.1126/sciadv.aee6417 (PMC13371933; doi:10.1126/sciadv.aee6417)
Supplement: Supplementary file 1 — Figs. S1 to S41 Notes S1 and S2 Table S1 Legends for movies S1 to S3 [file sciadv.aee6417_sm.pdf]

Science Advances
AAAS

Supplementary Materials for

Reduction of appearance artifacts in wearable on-skin electronics

Yijun Liu *et al.*

Corresponding author: Naoji Matsuhisa, naoji@iis.u-tokyo.ac.jp

Sci. Adv. **12**, eaee6417 (2026)
DOI: 10.1126/sciadv.aee6417

The PDF file includes:

Figs. S1 to S41
Notes S1 and S2
Table S1
Legends for movies S1 to S3

Other Supplementary Material for this manuscript includes the following:

Movies S1 to S3

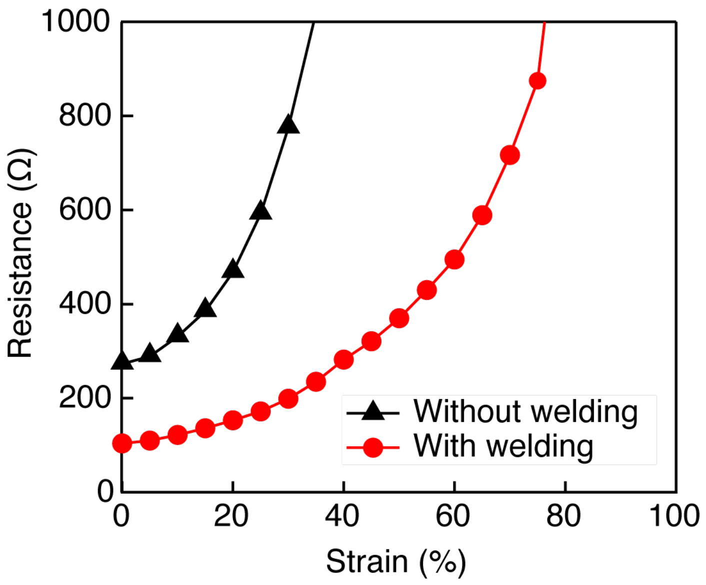

Fig. S1. Resistance-strain characteristics of AgNWs without welding treatment and with welding treatment.

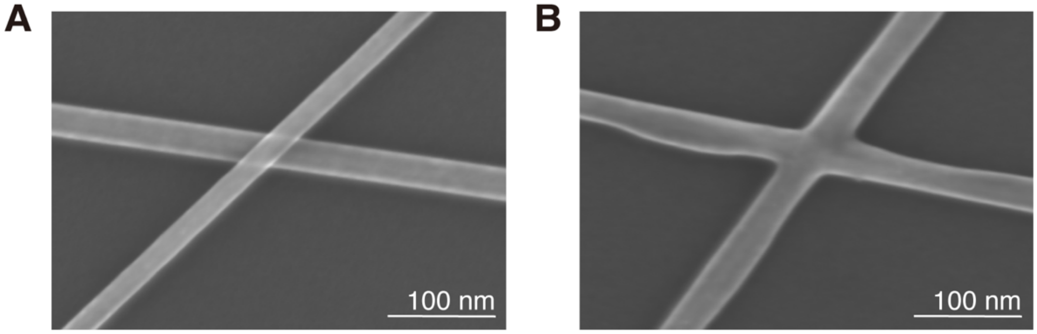

Fig. S2. SEM images of a junction between two silver nanowires, (A) before and (B) after welding.

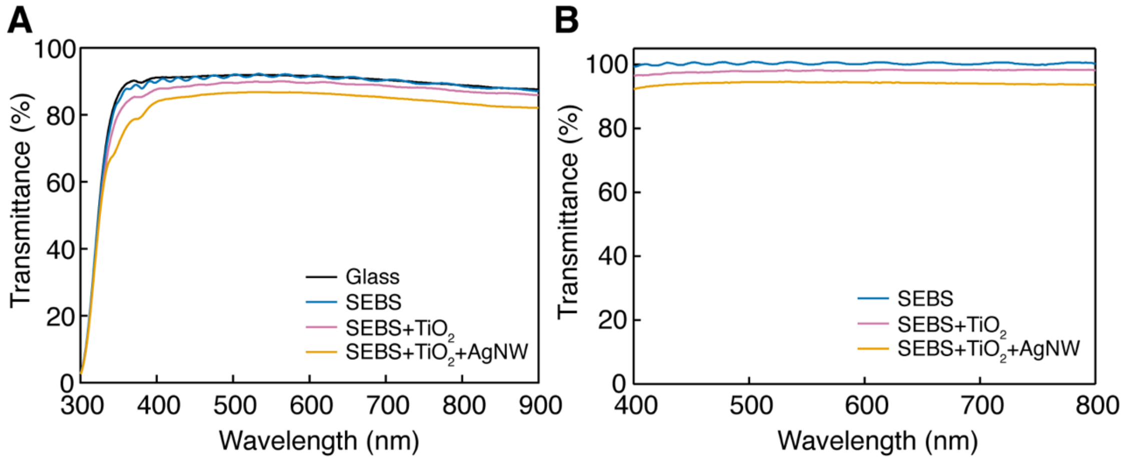

Fig. S3. UV–vis spectra. Optical transmittance spectra of the plain SEBS film, the optical adjustment layer (SEBS + TiO_2), and the invisible electrode (SEBS + TiO_2 + AgNWs). Transmittance spectra measured with air as reference **(A)** and with bare glass as reference **(B)**. The air-referenced spectrum includes substrate reflection losses, while the glass-referenced spectrum reveals the intrinsic transmittance of the electrode film exceeding 90% across the visible range.

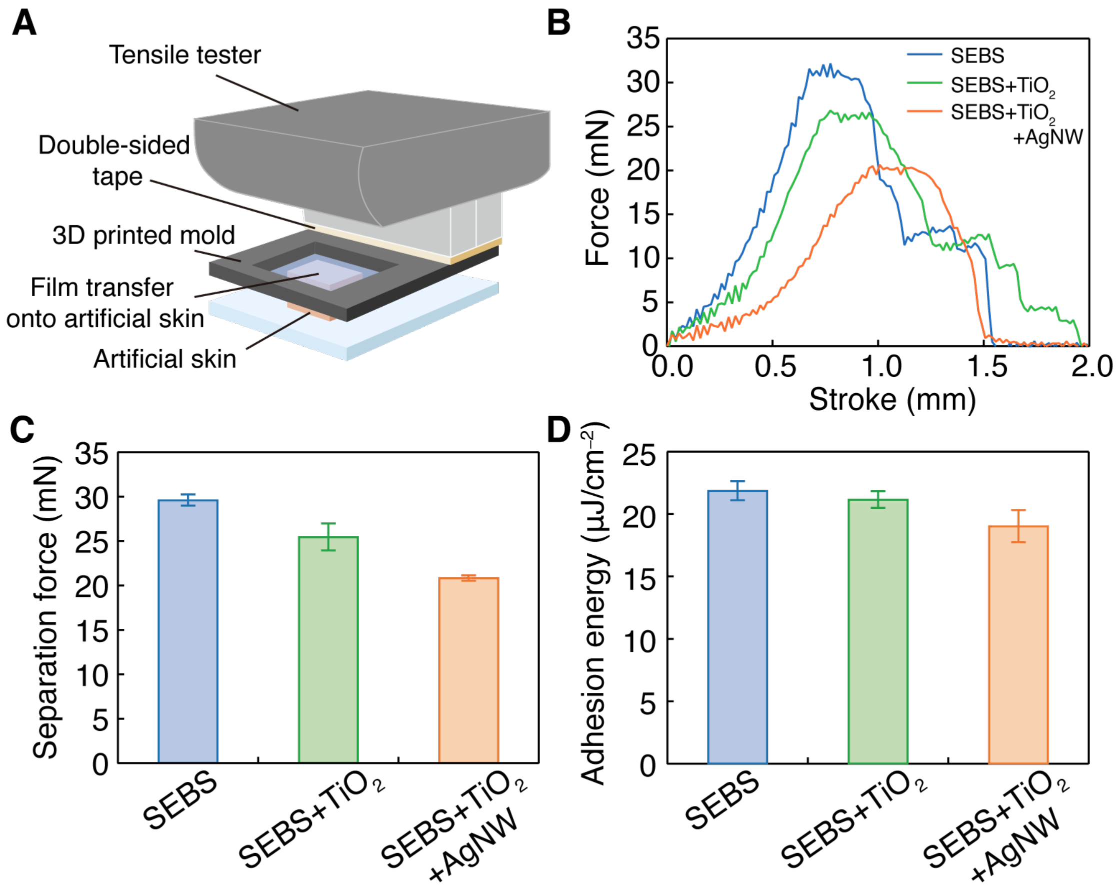

Fig. S4. Adhesion test. (A) A schematic of the setup. A 3D-printed mold with a 1.8 × 1.8 cm^2 window was used to support the samples. The film's edges were fixed to the window using strong double-sided tape, then the center part of the film was attached onto artificial skin on an area of 1 × 1 cm^2, using the same transfer procedure as described in the paper on human skin. Adhesion force was recorded using a tensile mechanical tester (Shimadzu, EZ-LX). The supporting window was lifted at a constant speed of 10 mm min^{-1} until complete detachment from the artificial skin. **(B)** Representative force–stroke curves of different film types: plain SEBS, SEBS+TiO_2, and SEBS+TiO_2+AgNW. **(C)** The separation force of the three types of film. n=4. **(D)** Comparisons of adhesion energy for the three types of film on artificial skin. n=4. Error bars denote standard error.

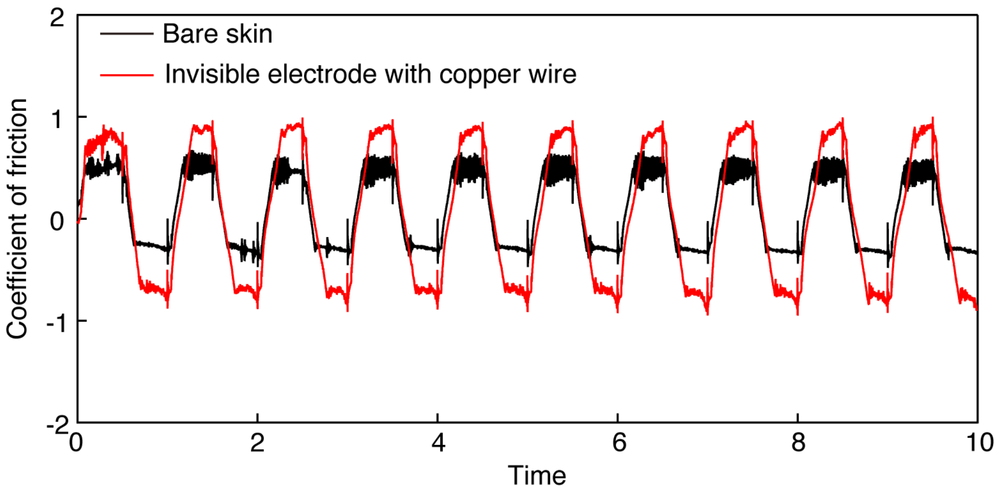

Fig. S5. Coefficient of friction measured on bare skin (black) and on the invisible electrode with copper wire (red) over ten repeated friction cycles. The invisible electrode keeps a stable friction pattern, indicating that it is robust and not easily damaged by rubbing.

Supplementary Note 1: Gloss

Gloss is used to describe the ability of an object's surface to reflect light. At the same angle of incidence, a high-gloss surface has a large specific reflection and looks bright. A low-gloss surface has less specific reflection and more diffuse reflection than a high-gloss surface, giving the object a matte appearance (Fig. S6). The addition of TiO_2 makes the film surface rough and the refractive index of the film uneven, reducing the gloss of the film.

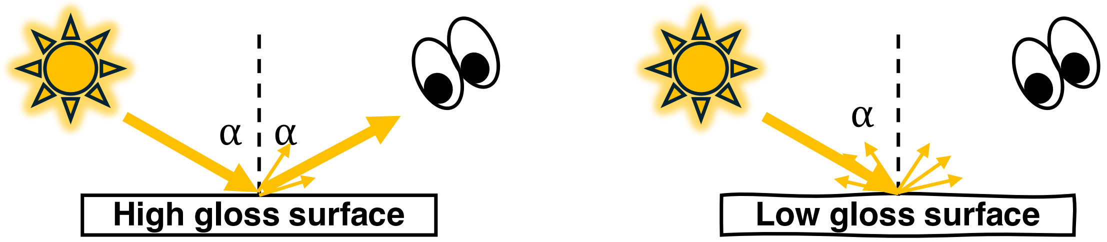

Fig. S6. A schematic to describe gloss.

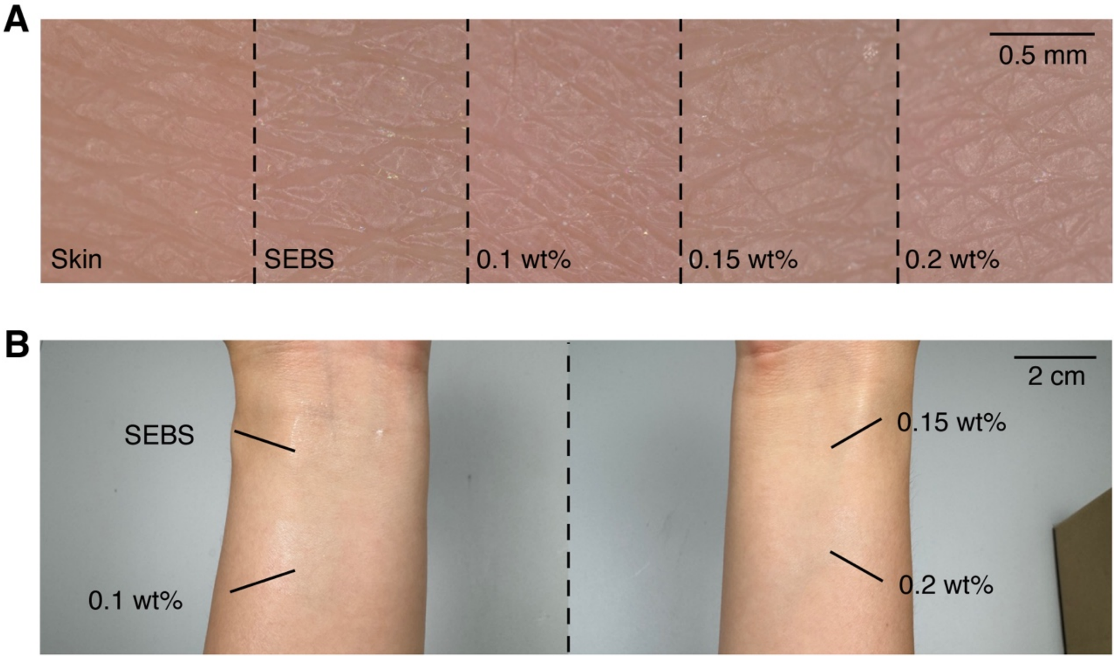

Fig. S7. Gloss of 200-nm-thick light scattering layers with different amounts of TiO_2 nanopowders. (A) Optical microscope images. **(B)** Photos of the films on skin under a D65 standard light source.

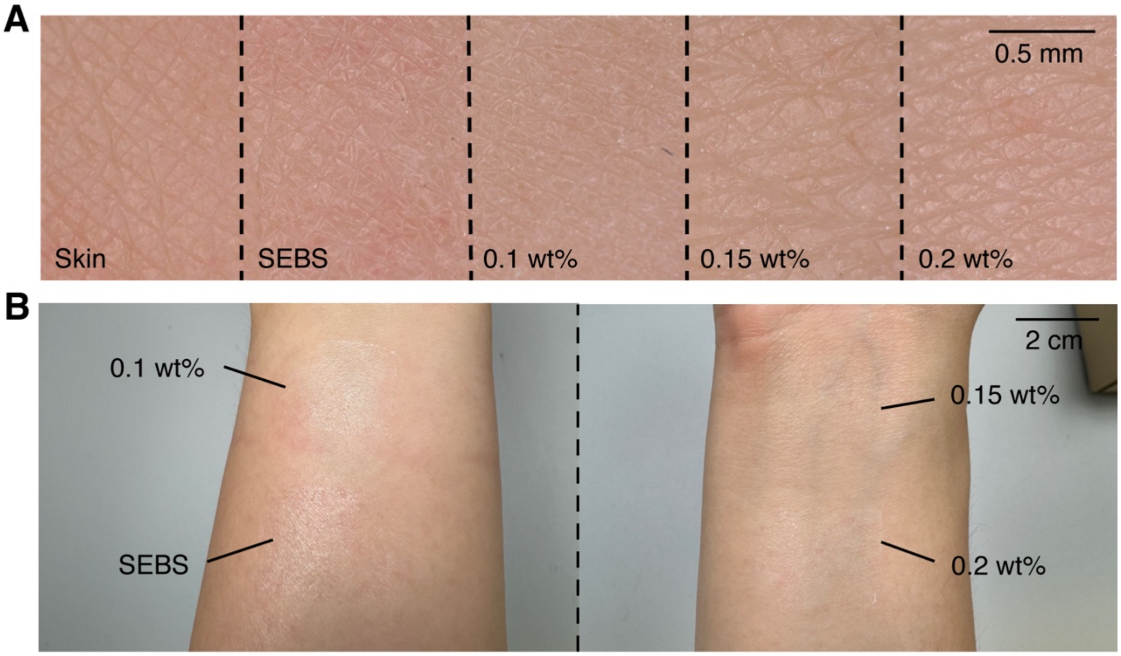

Fig. S8. Gloss of 400-nm-thick light scattering layers with different amounts of TiO_2 nanopowders. (A) Optical microscope images. **(B)** Photos of the films on skin under a D65 standard light source.

Supplementary Note 2: Diffuse reflection by TiO_2 nanopowders

The reflectance R of a thin film mainly depends on the difference in refractive index between the film and air. According to Fresnel's equation, when light perpendicularly enters two media with different refractive indices, the reflectance R is given by:

$$R = \left(\frac{n_1 - n_2}{n_1 + n_2}\right)^2$$

n_1: the refractive index of the incident medium

n_2: the refractive index of the thin film material.

TiO_2 nanopowder has a high refractive index (2.6-2.9) compared to that of SEBS (~1.5). TiO_2 nanopowders in SEBS reflect incident light in various directions to make diffuse reflection.

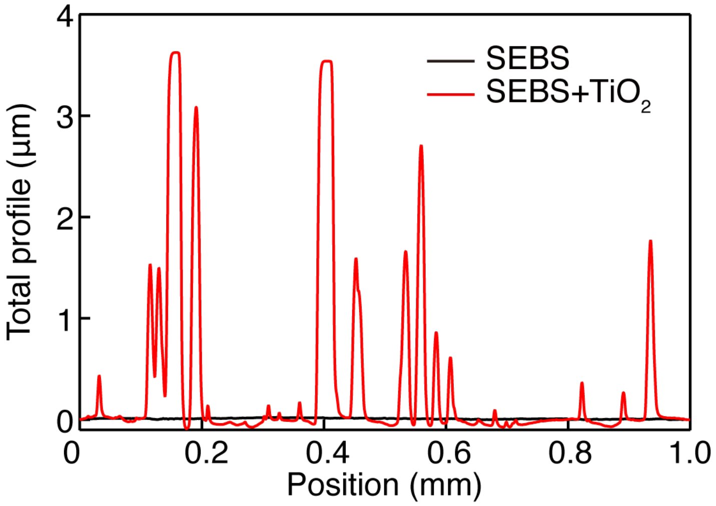

Fig. S9. Surface roughness of an optical adjustment layer observed by a stylus profiler.

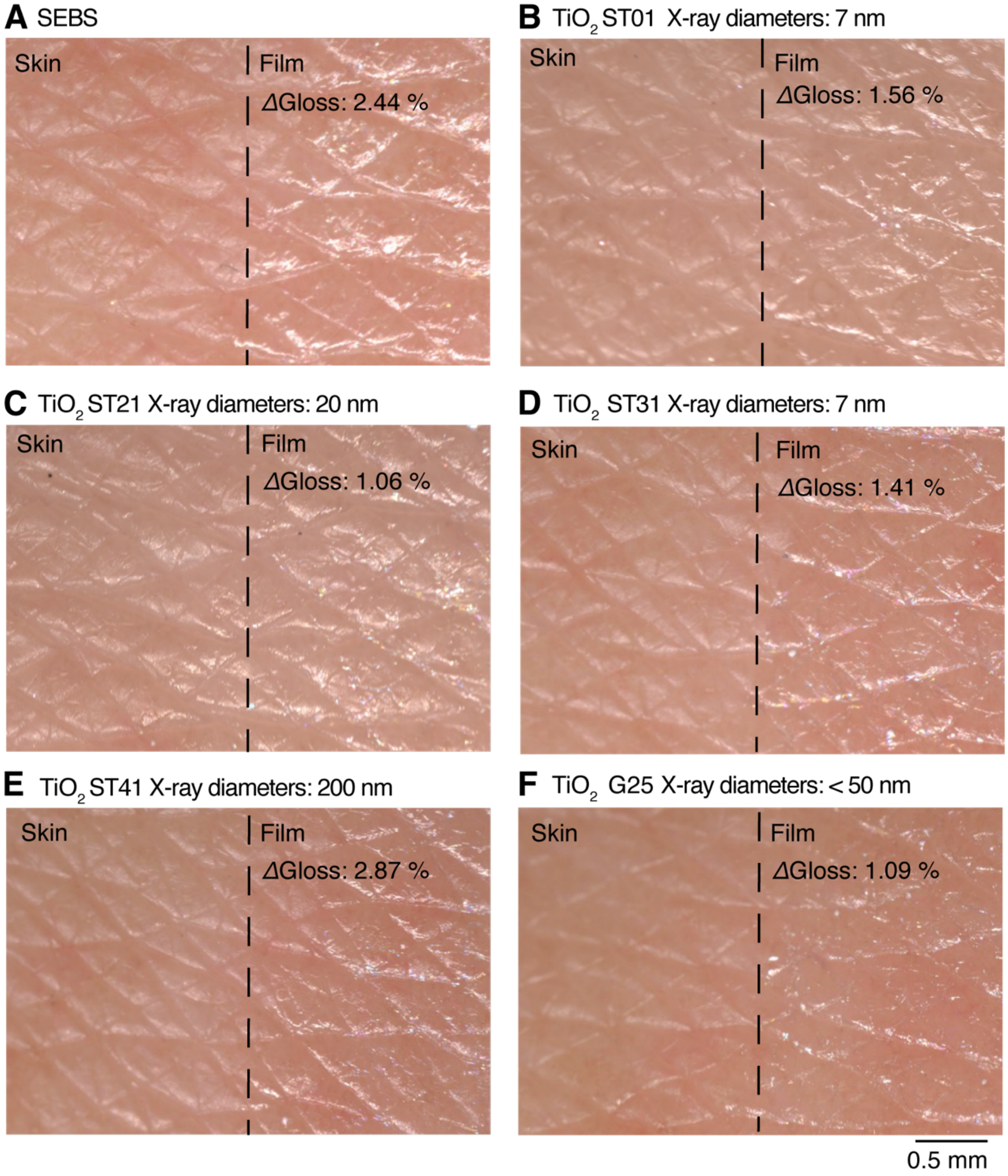

Fig. S10. Optical microscope image of the SEBS with different sizes of TiO_2. Optical microscope images comparing bare skin and **(A)** plain SEBS, and SEBS with TiO_2 nanopowders of different X-ray diameters **(B)** ST01, **(C)** ST21, **(D)** ST-31, **(E)** ST-41, and **(F)** G25. The left side shows bare skin, the right side shows film on skin. The gloss variation (ΔGloss) of each film is indicated in the top right. SEBS without TiO_2 exhibits a high gloss difference, while adding TiO_2 reduces ΔGloss. TiO_2 with sizes (~20-50 nm) exhibits the lowest gloss variation. Smaller particles (~7 nm) provide insufficient scattering to effectively suppress specular reflection, whereas larger particles (~200 nm) increase surface roughness and optical non-uniformity, leading to higher gloss differences.

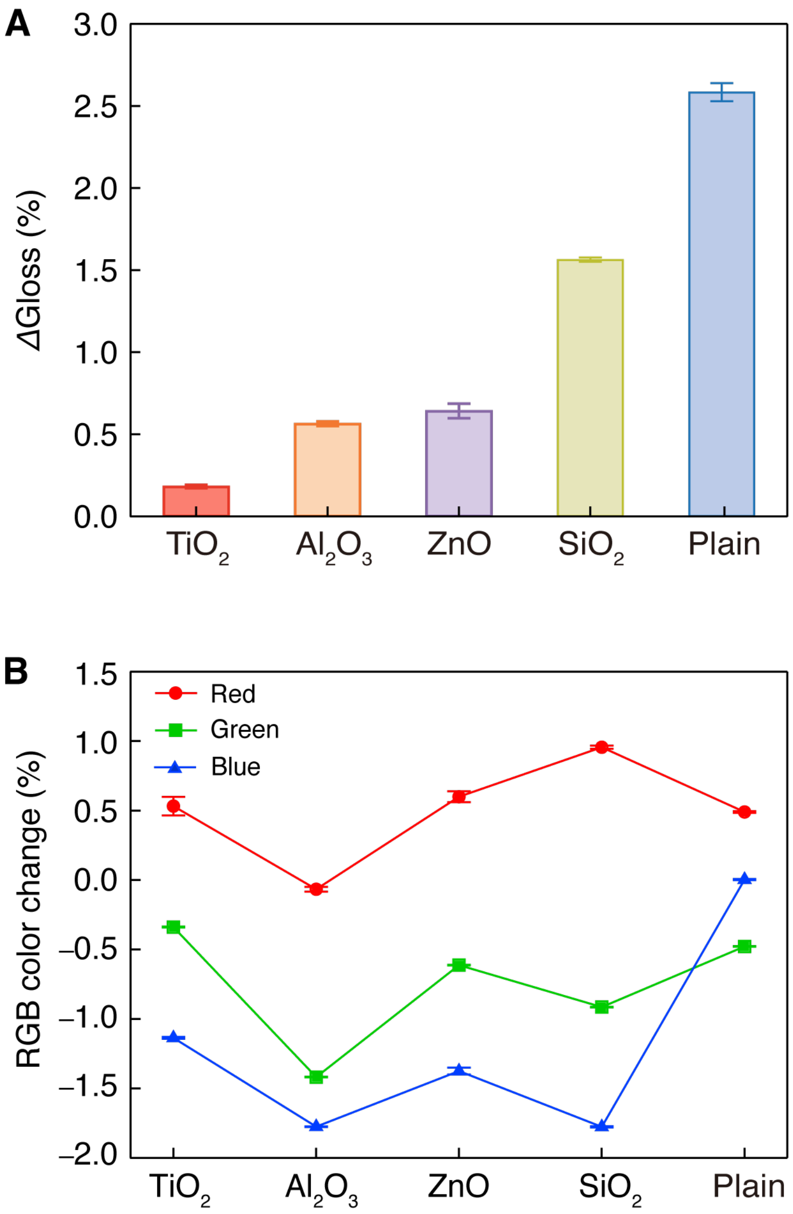

Fig. S11. Optical characteristics of thin films with different oxide nanopowders. (A) Change in gloss (ΔGloss) and **(B)** RGB color changes of films containing TiO_2 (particle size <100 nm), Al_2O_3 (particle size ~150 mesh, pore size: 58 Å), ZnO (particle size 20 nm), and SiO_2 (particle size 5-20 nm) at the same concentration (0.15 wt%) in SEBS. All films were fabricated with a thickness of 0.2 μm and attached to artificial skin for measurement. Compared with bare skin, TiO_2, Al_2O_3, ZnO, and SiO_2 all reduced gloss variation relative to the plain SEBS film, with TiO_2 showing the largest gloss reduction, indicating the most effective suppression of surface reflection. For all three RGB colors, the film with TiO_2 showed relatively small color changes compared with the other oxide nanopowders. Error bars denote standard error.

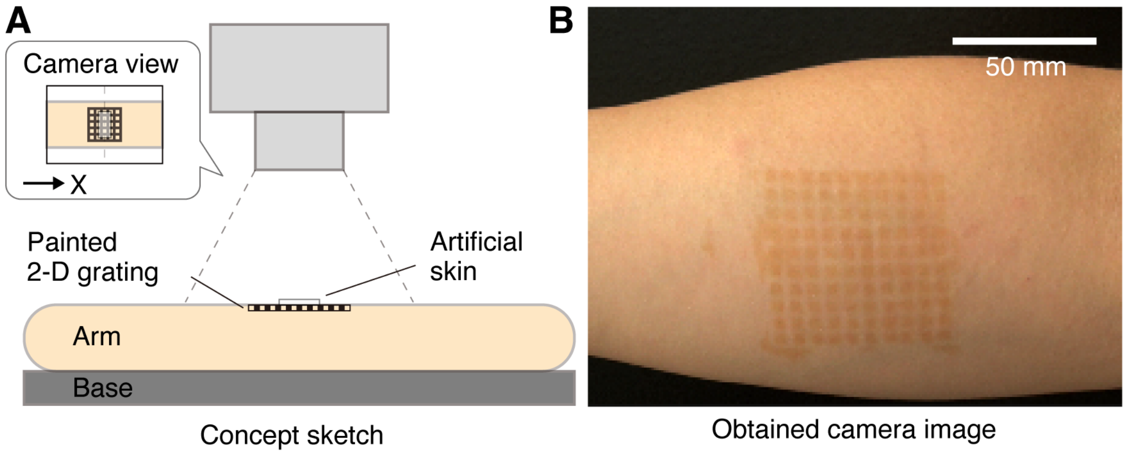

Fig. S12. Setup for measuring the compliance of the thin films under skin deformation. (A) Schematic illustration of the measurement setup. **(B)** An obtained image from the camera.

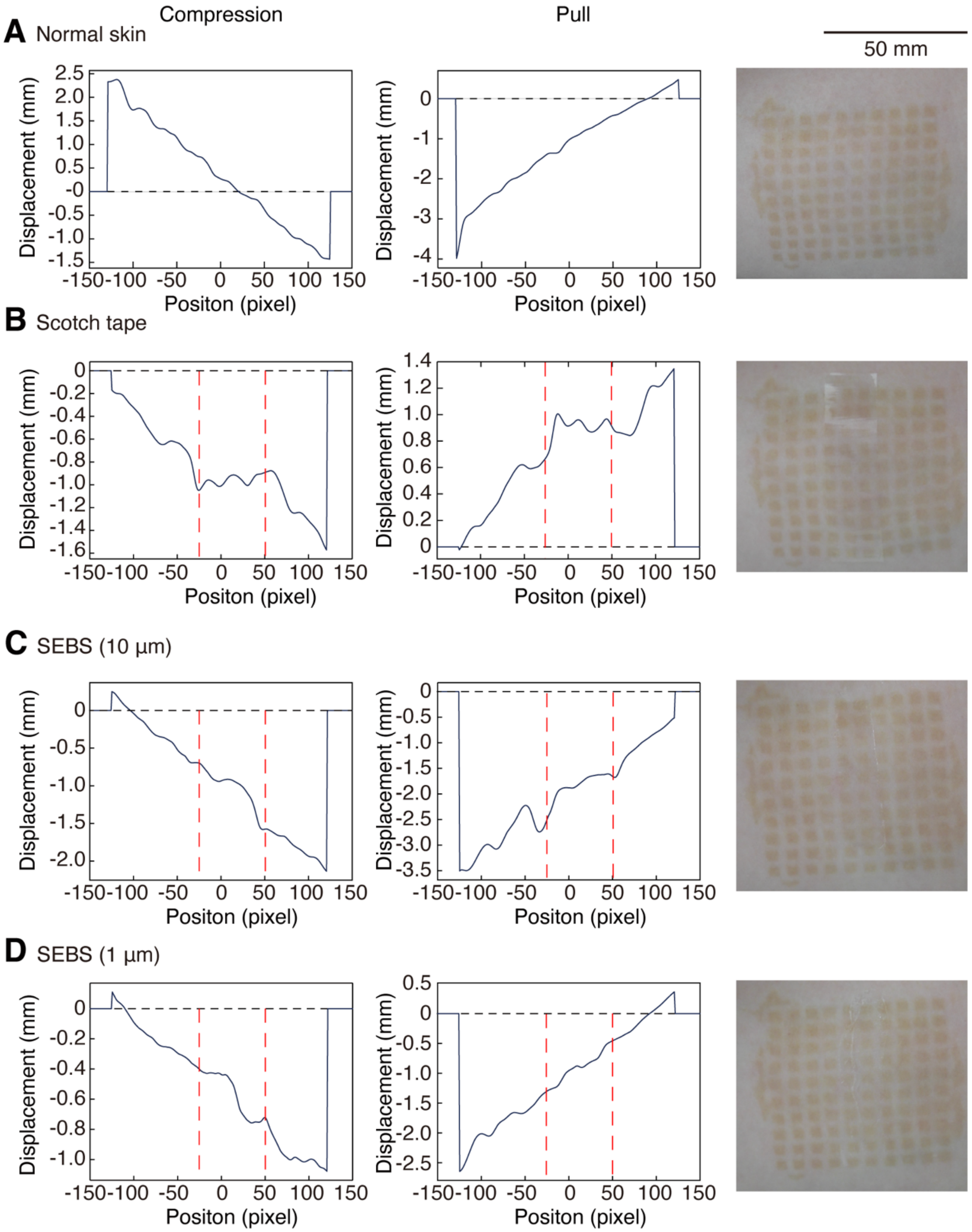

Fig. S13. Compliance to skin deformations. (A) Deformation of bare skin. The skin is linearly displaced in response to tension and compression. **(B)** Adhesive tape. The skin cannot freely deform in the area under the adhesive tape. **(C)** A 10-µm-thick SEBS film. The skin was linearly displaced even under the 10-µm-thick SEBS film. **(D)** A 1-µm-thick SEBS film. The skin displaced more linearly than in (C). Red dashed lines represent the different films' position on skin.

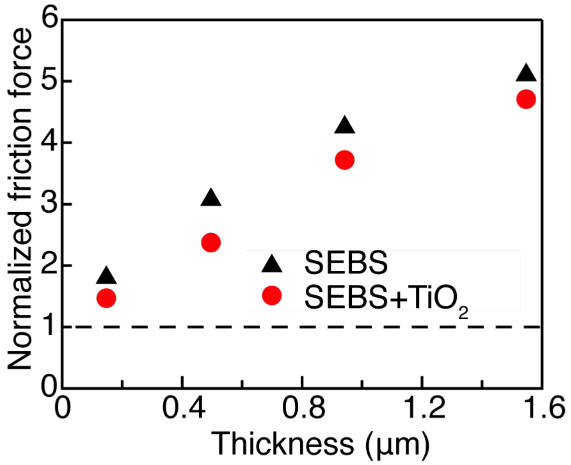

Fig. S14. Friction force of thin films on skin. The friction force is normalized by the measure on bare skin. Effect of films' thickness and TiO_2 (0.15 wt%) on normalized friction forces. Thin SEBS with TiO_2 showed friction forces closer to bare skin.

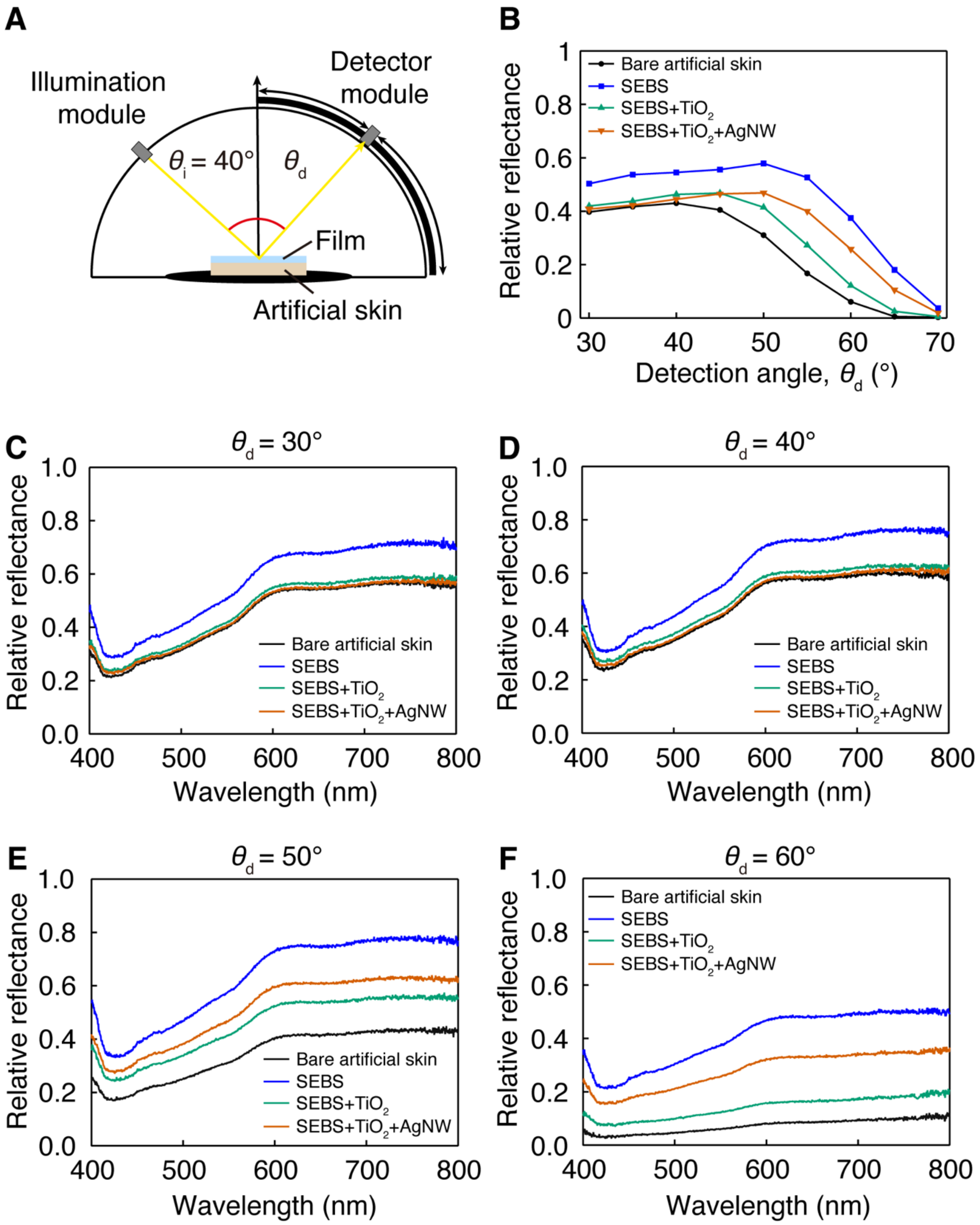

Fig. S15 Angle-dependent reflectance measurements. (A) Schematic of the angle-dependent reflectance measurement setup. The incident angle was 40°. A white A4 sheet of paper was used to obtain a reference reflectance. Light intensity was recorded at detection angles from 30° to 70°. Measurement was performed with bare artificial skin, plain SEBS, SEBS with TiO_2, AgNW-coated SEBS with TiO_2. All the film thickness was 200 nm. **(B)** Comparison of relative reflectance at detection angles from 30° to 70°. **(C-F)** Reflectance spectra measured at detection angles of 30° **(C)**, 40° **(D)**, 50° **(E)**, and 60° **(F)**.

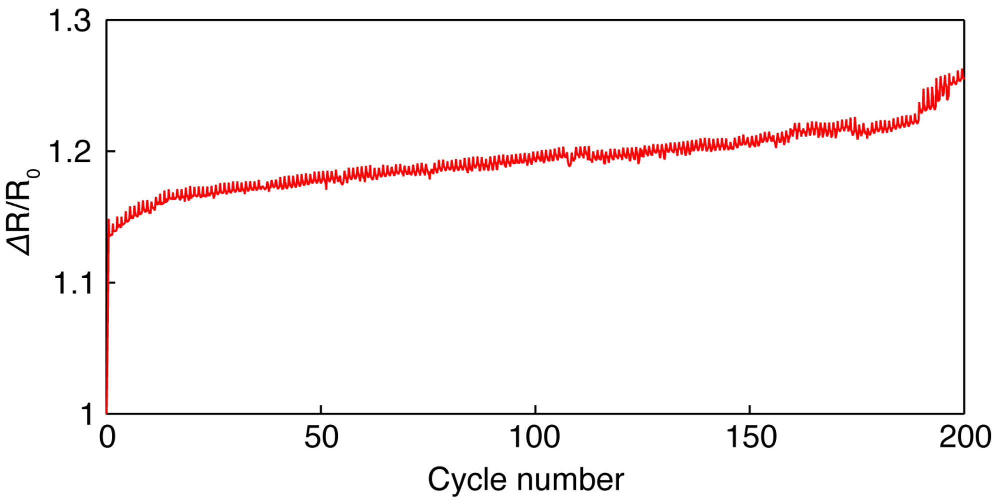

Fig. S16. Electrical stability under repeated mechanical deformation. The invisible electrode was transferred onto artificial skin with an initial length of 1 cm and subjected to repeated stretching at a strain of 20%. The displacement was applied at a speed of 0.5 mm s^{-1}. The electrical resistance was continuously recorded during cyclic deformation to demonstrate stable electrical performance under repeated deformation.

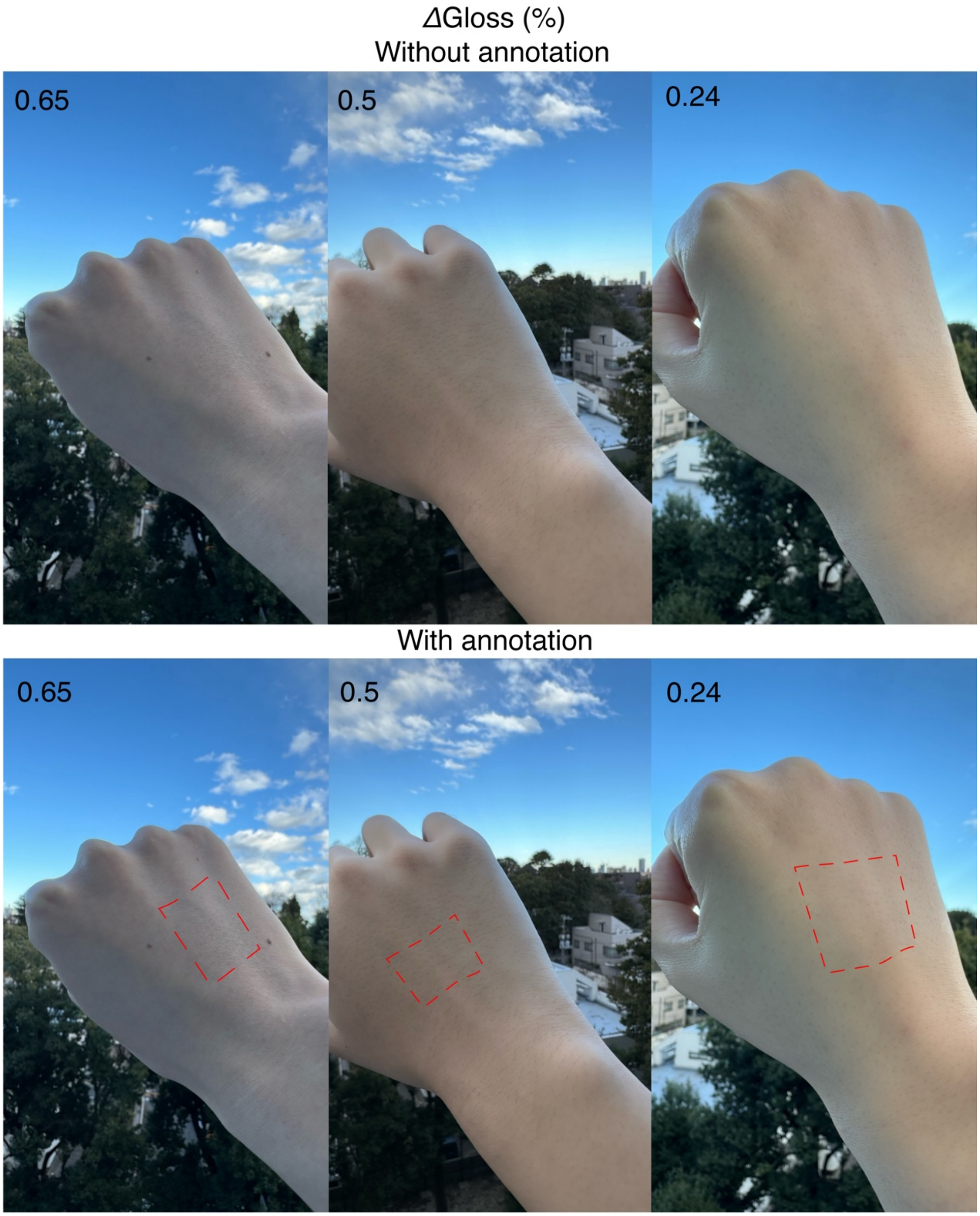

Fig. S17. Photographs of the invisible electrode on the skin of different volunteers under natural sunlight.

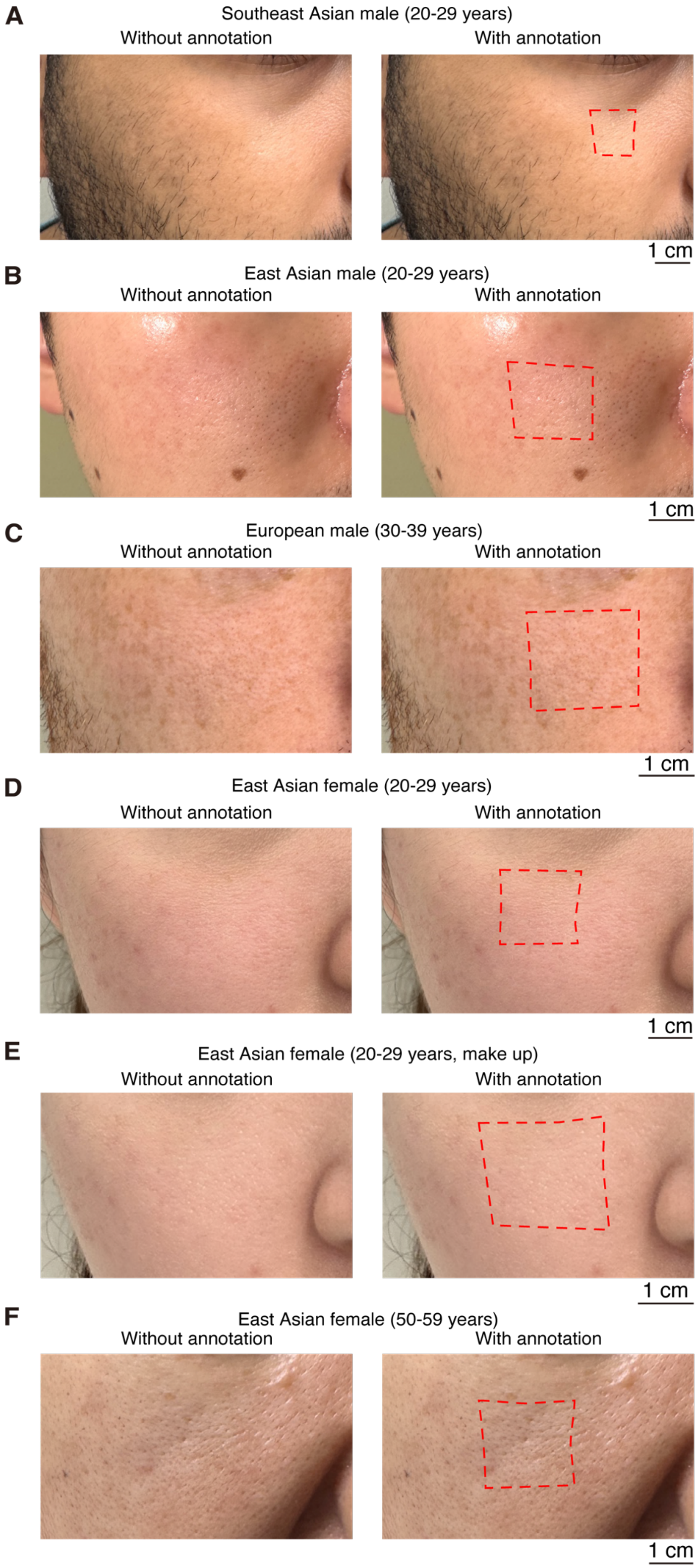
A
Southeast Asian male (20-29 years)
Without annotation
With annotation
1 cm
B
East Asian male (20-29 years)
Without annotation
With annotation
1 cm
C
European male (30-39 years)
Without annotation
With annotation
1 cm
D
East Asian female (20-29 years)
Without annotation
With annotation
1 cm
E
East Asian female (20-29 years, make up)
Without annotation
With annotation
1 cm
F
East Asian female (50-59 years)
Without annotation
With annotation
1 cm

Fig. S18 Optical invisibility of electrodes on different skin types. Photographs of the invisible electrode attached to the faces of volunteers with diverse skin tones and age groups: (A) Southeast Asian male aged 20–29 years, (B) East Asian male aged 20–29 years, (C) European male aged 30–39 years, (D) East Asian female aged 20–29 years, (E) East Asian female aged 20–29 years wearing makeup, and (F) East Asian female aged 50–59 years. The invisible electrode exhibits excellent invisibility across all skin types, seamlessly blending with the surrounding skin regardless of skin tone or age-related variations.

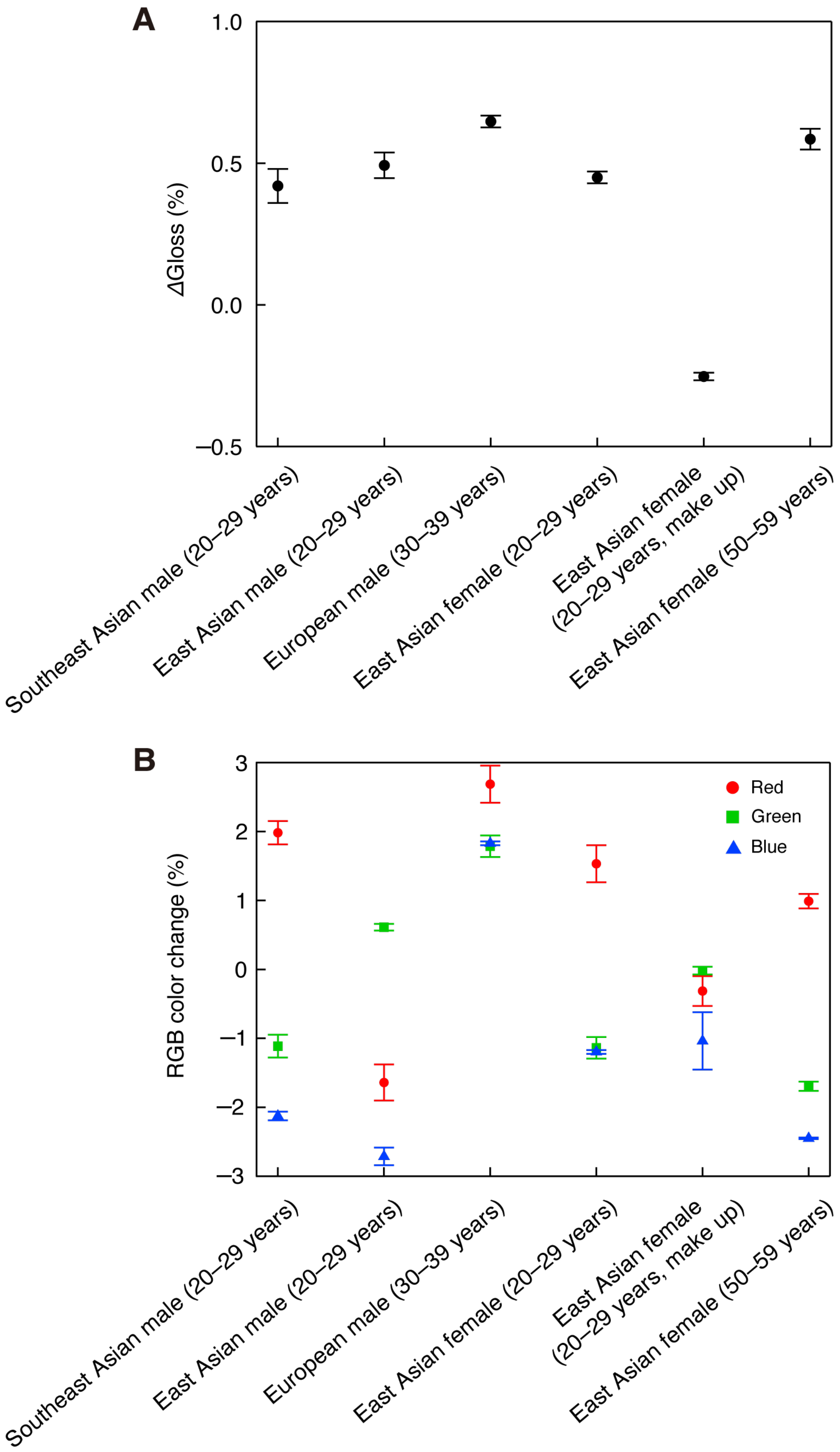

A
1.0
0.5
0.0
−0.5
ΔGloss (%)
Southeast Asian male (20–29 years)
East Asian male (20–29 years)
European male (30–39 years)
East Asian female (20–29 years)
East Asian female (20–29 years, make up)
East Asian female (50–59 years)
B
3
2
1
0
−1
−2
−3
RGB color change (%)
Red
Green
Blue
Southeast Asian male (20–29 years)
East Asian male (20–29 years)
European male (30–39 years)
East Asian female (20–29 years)
East Asian female (20–29 years, make up)
East Asian female (50–59 years)

Fig. S19 Gloss and RGB color variation across different skin types. (A) Gloss measurements comparing the invisible electrode and bare skin for all volunteer groups showed minimal differences across all skin types, with values below 0.7%. For the makeup group (East Asian female), the gloss variations became slightly negative, as commercial foundation containing brightening components increased skin gloss while TiO_2 in the electrode suppressed this reflection. **(B)** RGB value changes between the electrode and bare skin. The color difference remained below 3% for all volunteers across different ethnic and age groups. Error bars denote standard error.

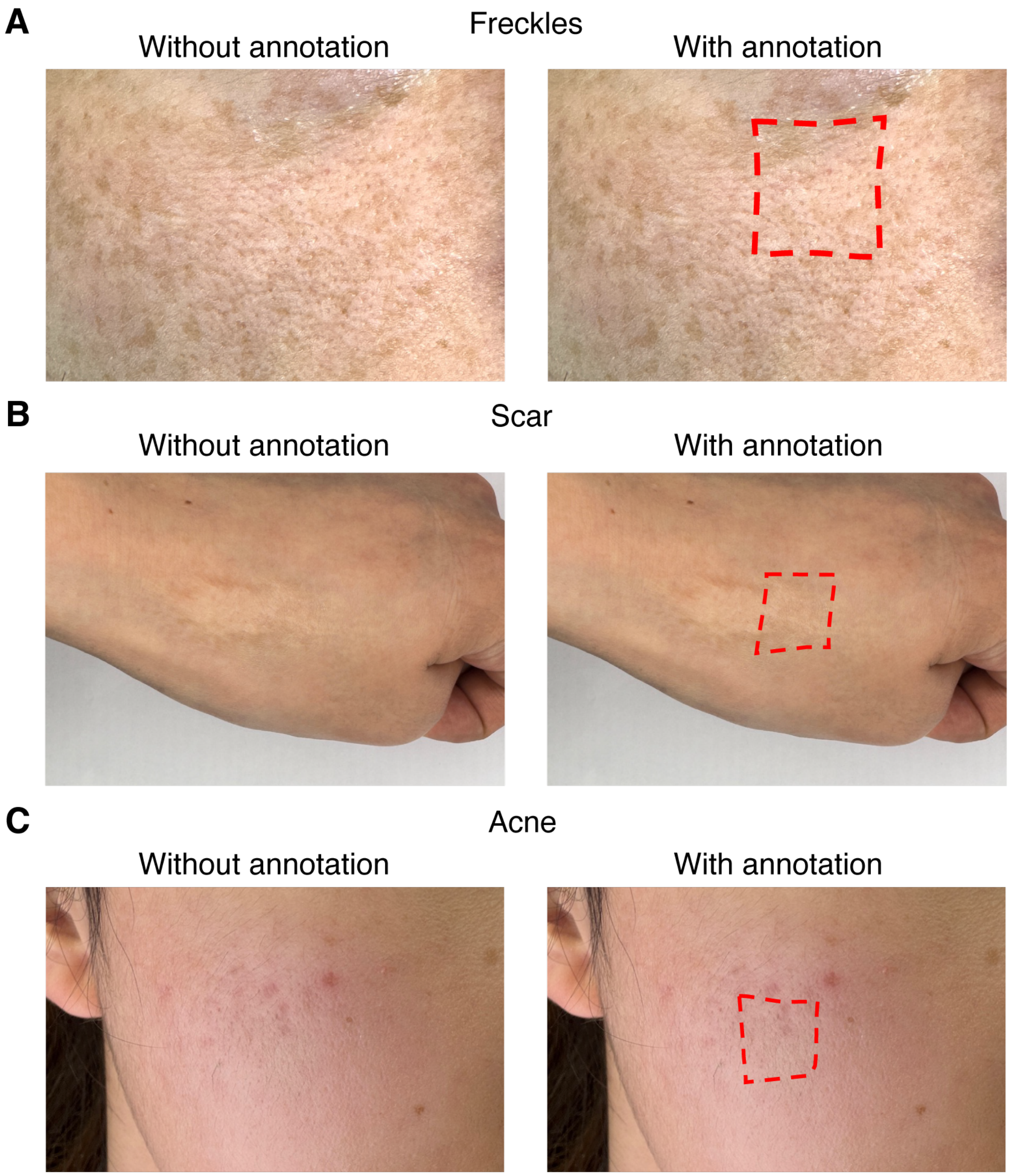

Fig. S20. Photos of invisible electrodes on different skin regions. (A) Freckles, **(B)** Scar and **(C)** Acne.

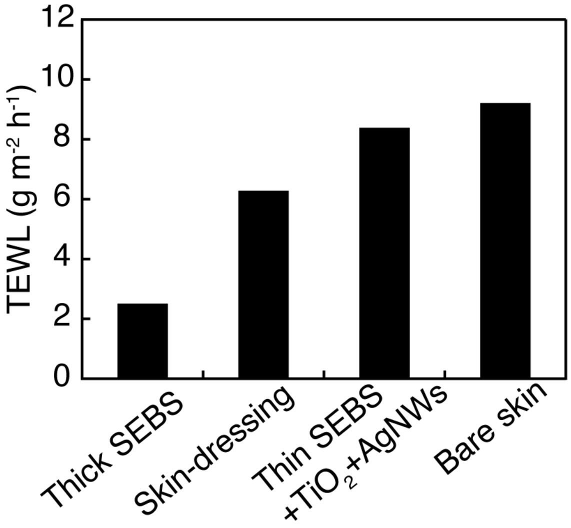

Fig. S21. TEWL values for different films attached on skin.

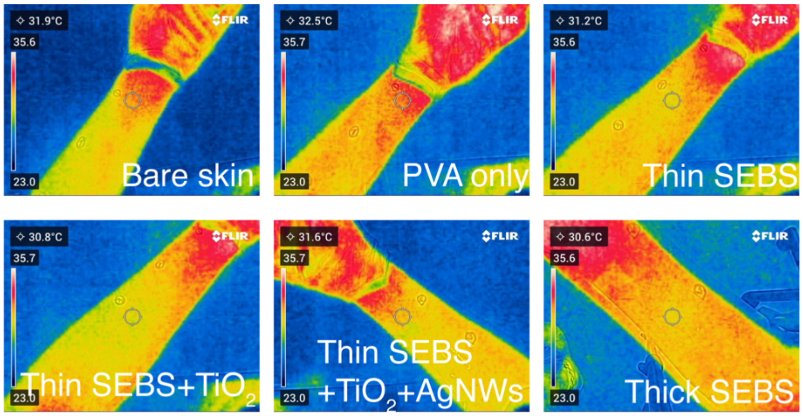

Film	Epidermal temperature change
PVA only	0.7 ^{o}C
Thin SEBS	1 ^{o}C
Thin SEBS+TiO_2	0.9 ^{o}C
Thin SEBS+TiO_2+AgNWs	0.2 ^{o}C
Thick SEBS	0.5 ^{o}C

Fig. S22. Thermal impact of thin films on skin. IR camera measurements of the epidermal temperature of different films on skin. The table records the epidermal temperature variation.

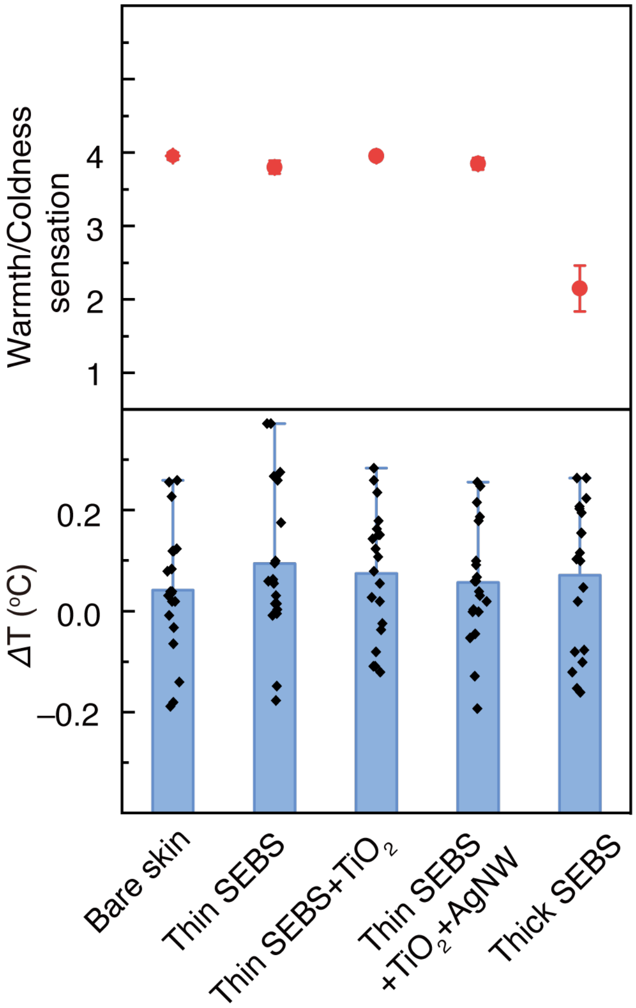

Fig. S23. Warmth/coldness sensation. Wearers subjectively evaluated the warmth/coldness sensation by each film (upper graph). Epidermal temperature change was simultaneously measured for each film (lower graph). Thick SEBS film, which showed significant differences (p-value less than 0.005) in all evaluations compared to the control group, is not specially marked. Error bars denote standard error.

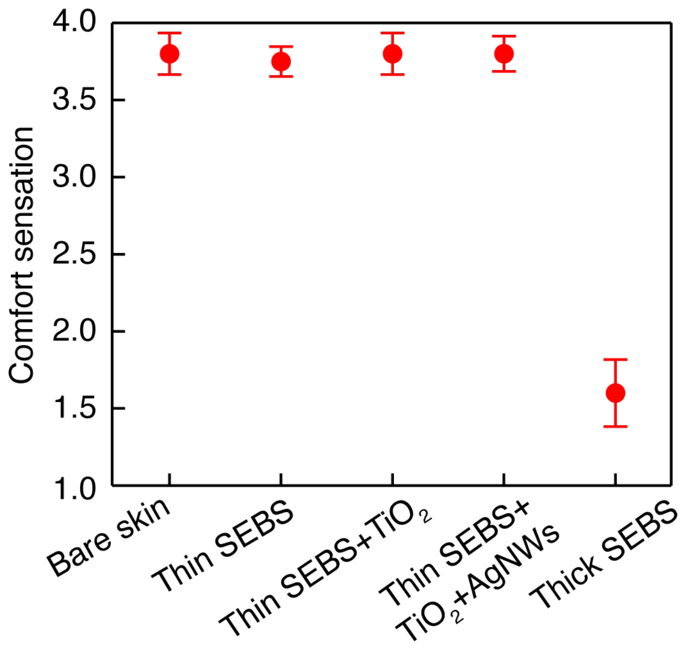

Fig. S24. Volunteers subjectively evaluated the comfort sensation by each film. Error bars denote standard error.

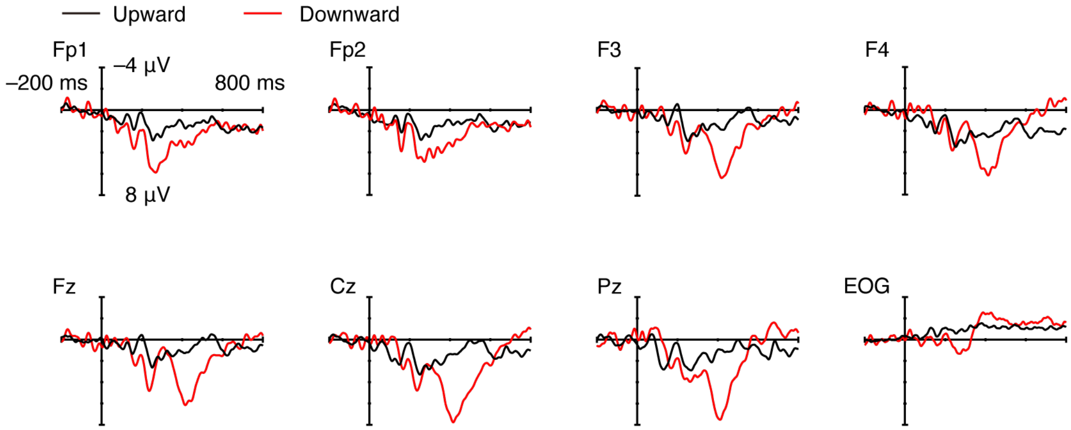

Fig. S25. Result of the Odd-ball task. Electrodes were placed following the 10/20 system at eight positions— front polar (Fp1, Fp2), frontal (F3, F4), midline frontal (Fz), the central (Cz), midline parietal (Pz), and below the left eye—for EEG and EOG signal recording. The reference electrode was located at the left earlobe, and the ground was located at the right earlobe. A screen in front of volunteers showed an upward or downward triangle randomly. Volunteers were asked to press a button when an upward triangle appeared. The graphs show the grand-average ERP waveforms for the upward triangle (black) and the downward triangle (red).

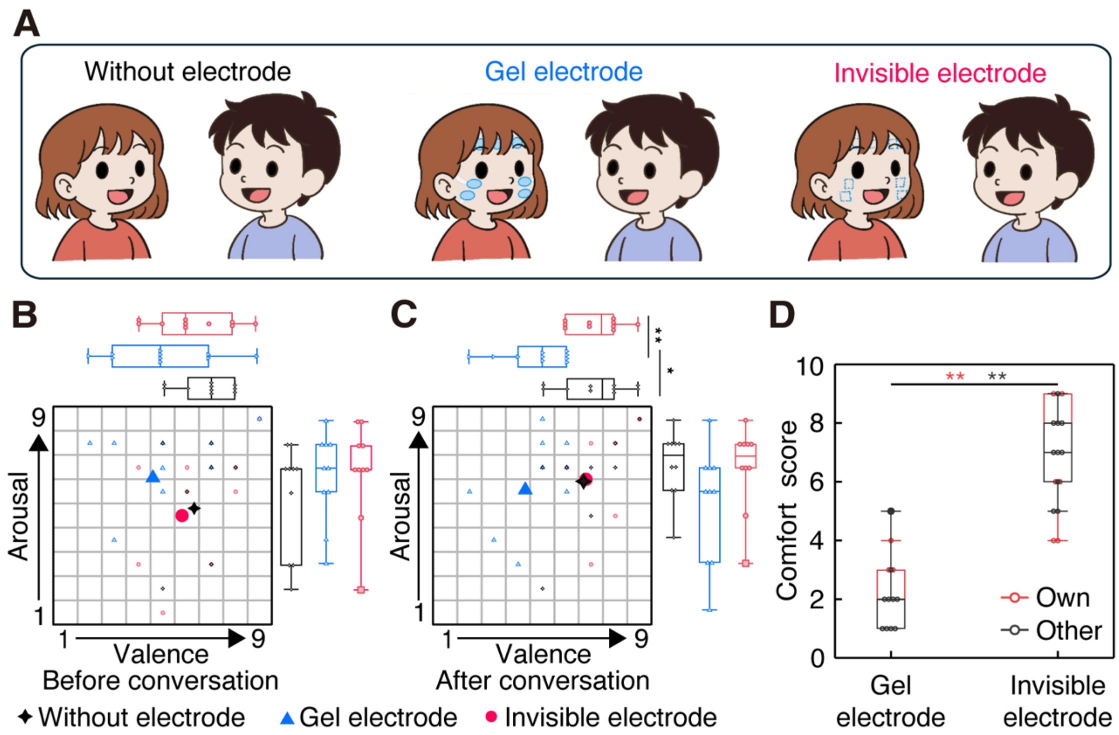

Fig. S26. A psychological experiment to assess the influence of the visibility of the electrodes during conversations. (A) Schematic diagram of the experiment conditions (created by Yijun Liu). Volunteers wore electrodes (without electrode (left), gel electrode (middle), and invisible electrode (right)) during the free conversation. Affect Grid plots of valence (horizontal axis) and arousal (vertical axis) before **(B)** and after **(C)** conversation. Box plots adjacent to the grids show the distributions of valence (top) and arousal (right) scores for each electrode condition. Non-parametric tests demonstrated significant differences in valence after conversation between the "without electrode" and "gel electrode" conditions (*$P < 0.05$) and between the "invisible electrode" and "gel electrode" conditions (**$P < 0.01$). **(D)** Questionnaire results reflecting the psychological comfort of volunteers and their partners during conversations when volunteers wore the invisible electrode or the gel electrode. A significant difference was observed between the invisible electrode and gel electrode conditions (**$P < 0.01$).

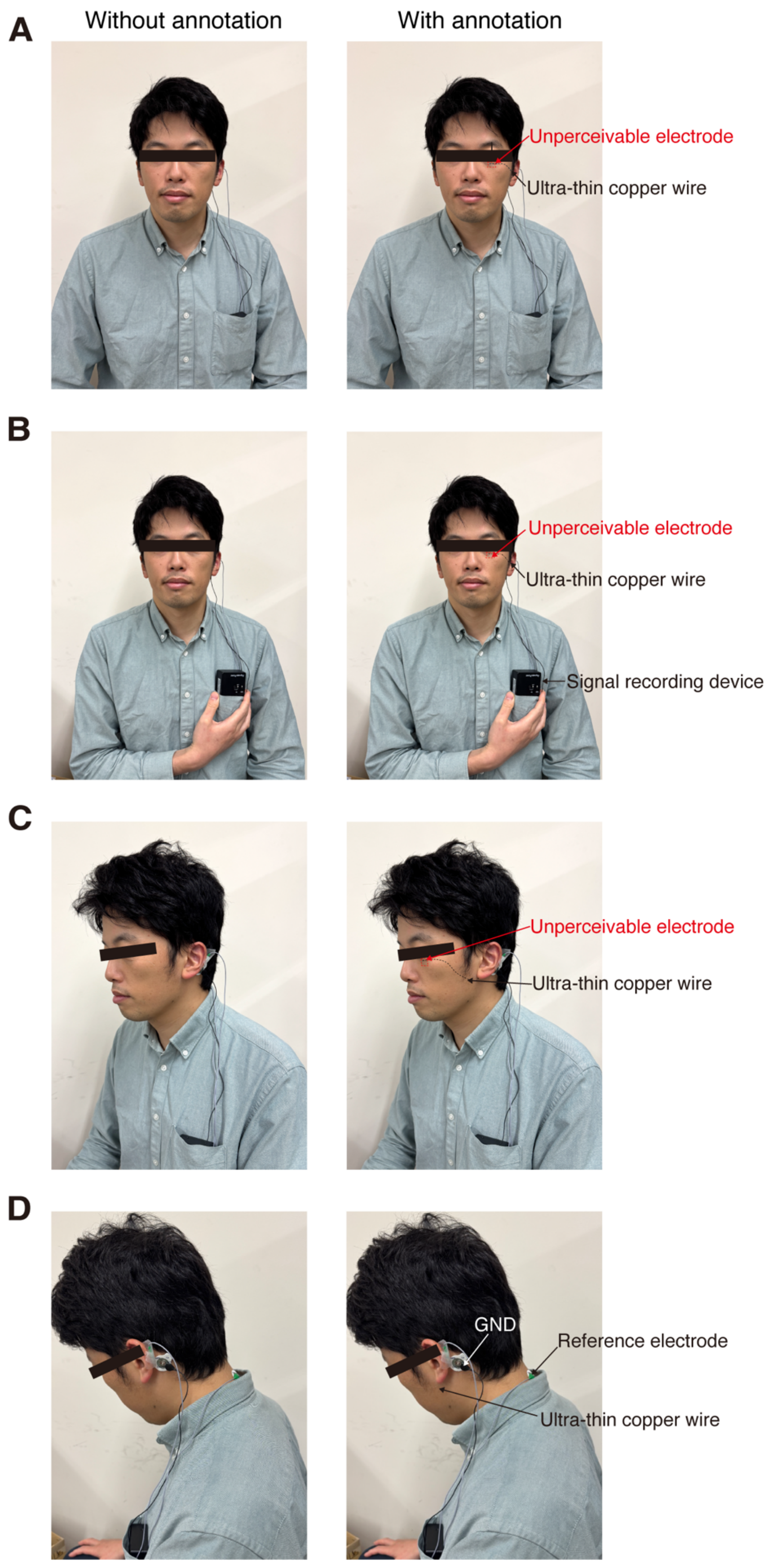
A
Without annotation
With annotation
Unperceivable electrode
Ultra-thin copper wire
B
Unperceivable electrode
Ultra-thin copper wire
Signal recording device
C
Unperceivable electrode
Ultra-thin copper wire
D
GND
Reference electrode
Ultra-thin copper wire

Fig. S27. The images of the full system from various viewing angles, including (A) front, (B) holding the recording device, (C) lateral, and (D) rear views, respectively. The left images show without annotation. The right images show with annotation indicating the positions of the invisible electrode and ultra-thin copper wire. The reference electrode and ground electrode were placed in vertebra prominens and mastoid process.

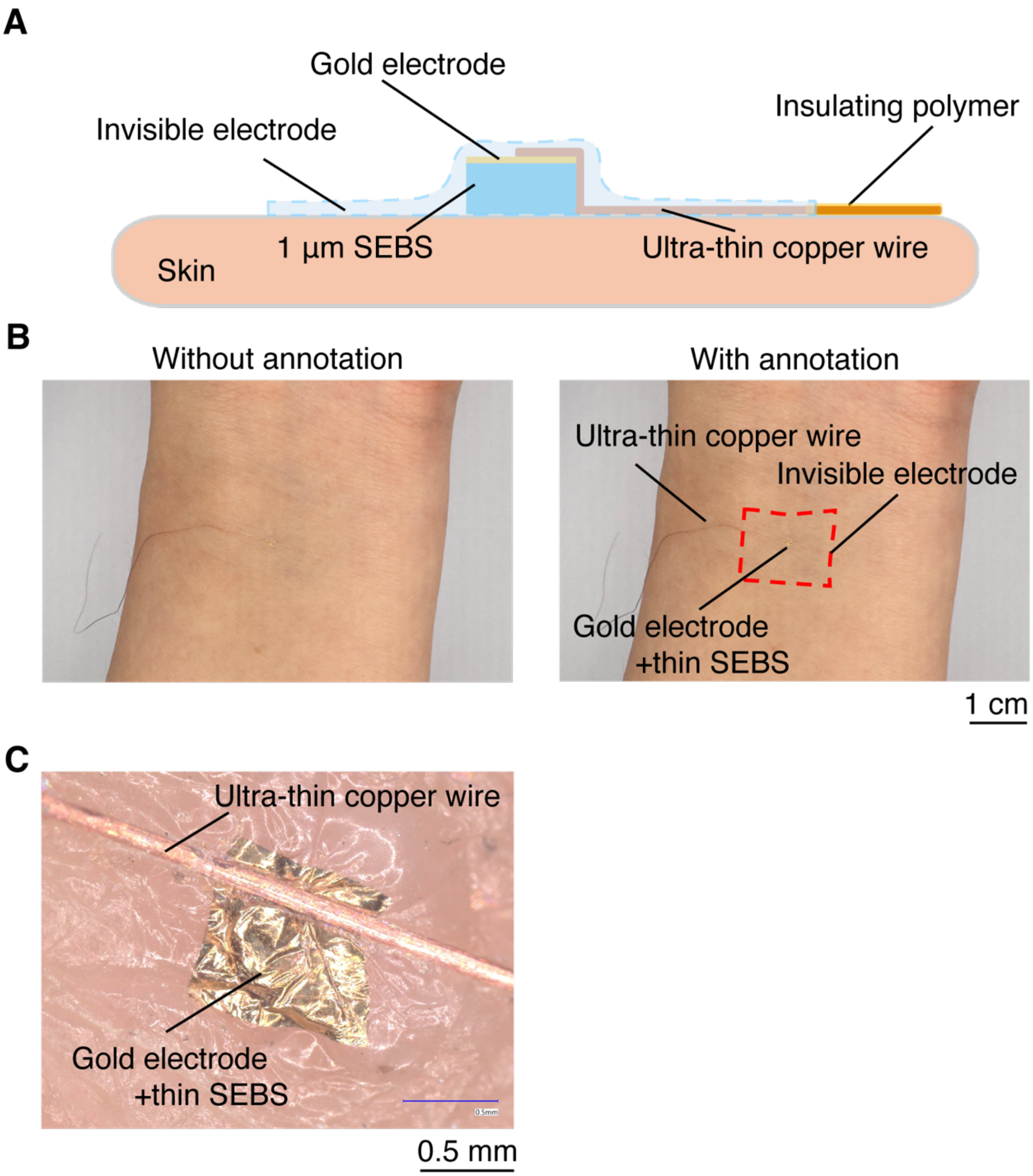

Fig. S28. On-skin integration of the invisible electrode. (A) Schematic illustration of the invisible device structure. **(B)** Photographs of the device on skin, shown without and with annotation. **(C)** Optical microscope image of the electrode–skin interface, showing conformal contact between the device and the skin microtexture. Scale bar, 0.5 mm.

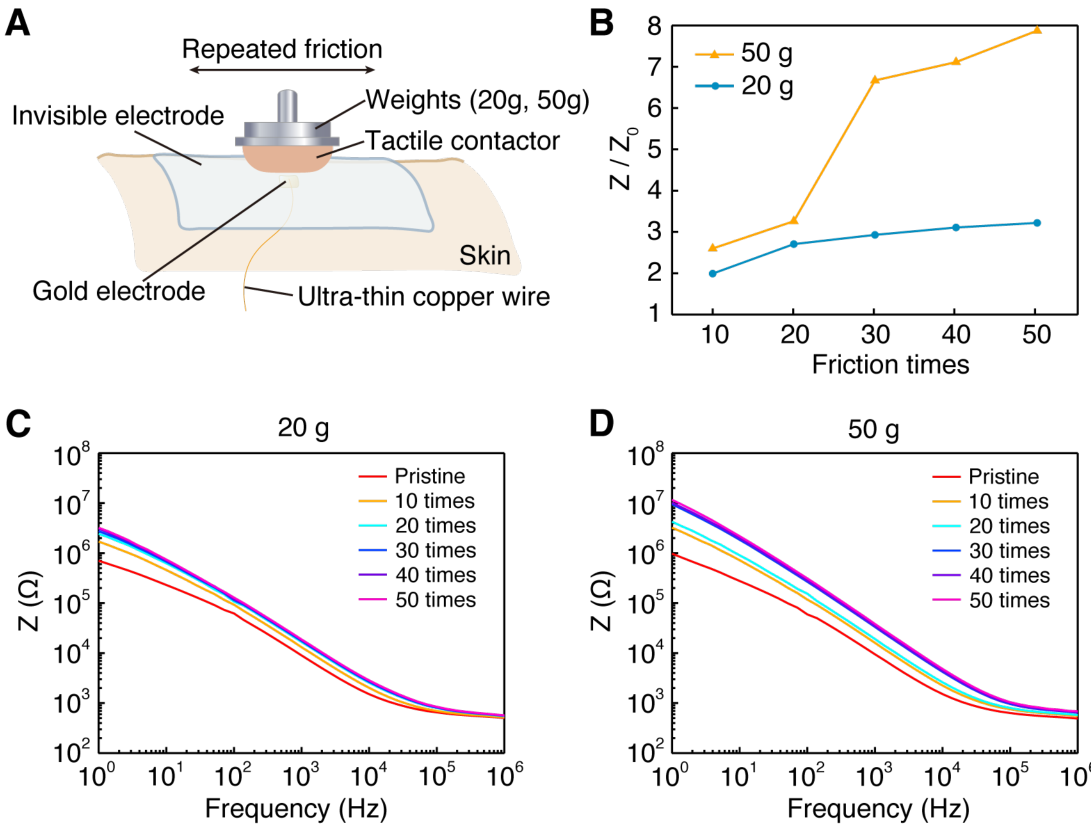

Fig. S29. Stability of electrical performance of the invisible electrode under repeated friction. (A) Schematic of the friction test. The tactile contactor (finger model) was placed horizontally on the electrode surface. Friction was applied over a sliding distance of 20 mm at a speed of 10 mm s^{-1} under controlled normal loads of 20 g and 50 g. The invisible electrode was attached to the volunteer's arm, and a reference electrode (gel electrode) of the same area was attached 5 cm away from the invisible electrode. **(B)** Relative impedance change (Z/Z_0) as a function of friction cycles under different applied loads (20 g and 50 g). The impedance gradually increases with friction cycles under both loading conditions, with a larger increase at 50 g, while the electrode remains within a functional range for stable electrical performance. Frequency-dependent skin impedance spectra measured under applied loads of 20 g **(C)** and 50 g **(D)**.

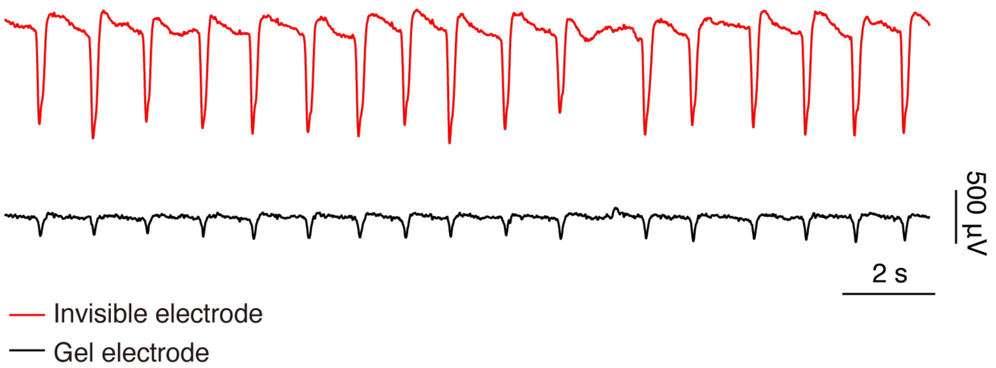

Fig. S30. Part of the EOG blink signal used for statistical analysis. The gel electrode was placed under the volunteer's left eyelid, and the invisible electrode was placed under the right eyelid. The reference electrode and ground electrode were placed in vertebra prominens and mastoid process, respectively. The volunteer was guided to blink both eyes simultaneously, and 150 blink events were recorded.

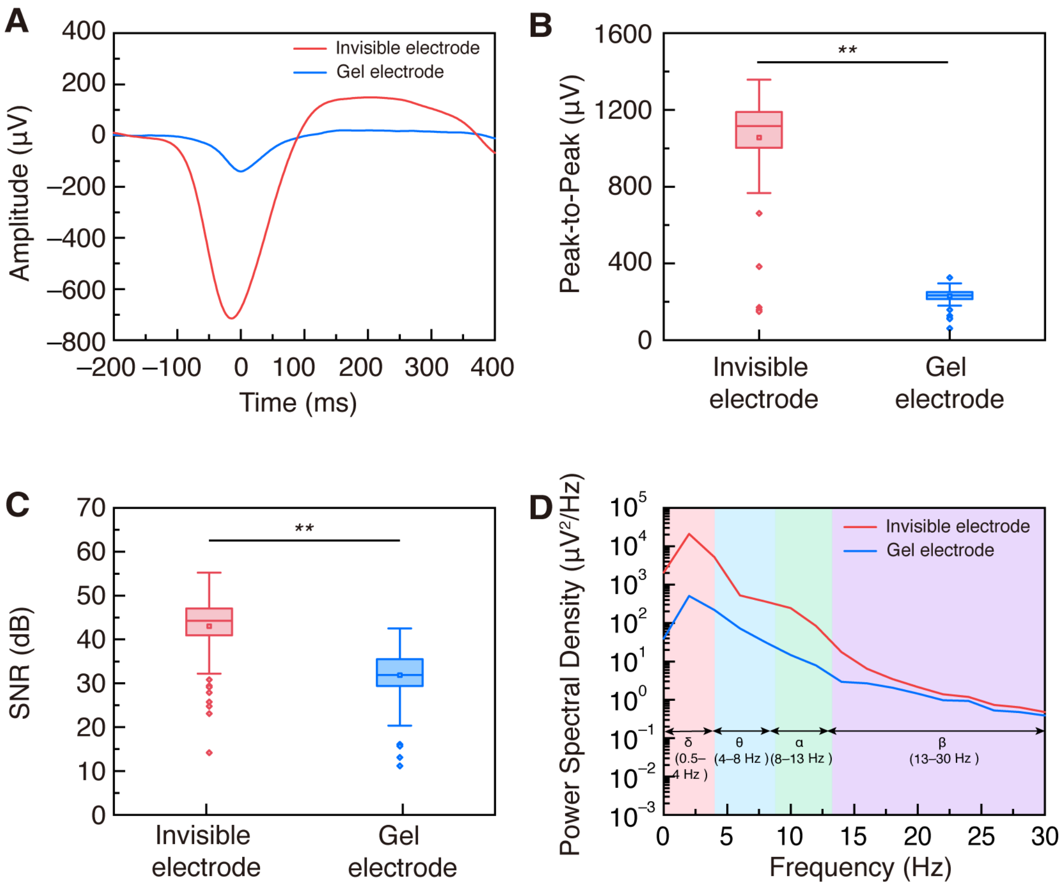

Fig. S31. Comparison of EOG signal quality between invisible and commercial gel electrodes. **(A)** The averaged blink waveforms were recorded using invisible and gel electrodes. **(B)** Peak-to-peak (P–P) amplitudes of blink signals recorded using invisible and gel electrodes, which showed significant differences ($P^{**} < 0.01$). **(C)** Signal-to-noise ratio (SNR) comparison of invisible and gel electrode ($P^{**} < 0.01$). **(D)** Power spectral density (PSD) analysis within 0–30 Hz, covering δ (0.5–4 Hz), θ (4–8 Hz), α (8–13 Hz), and β (13–30 Hz) bands.

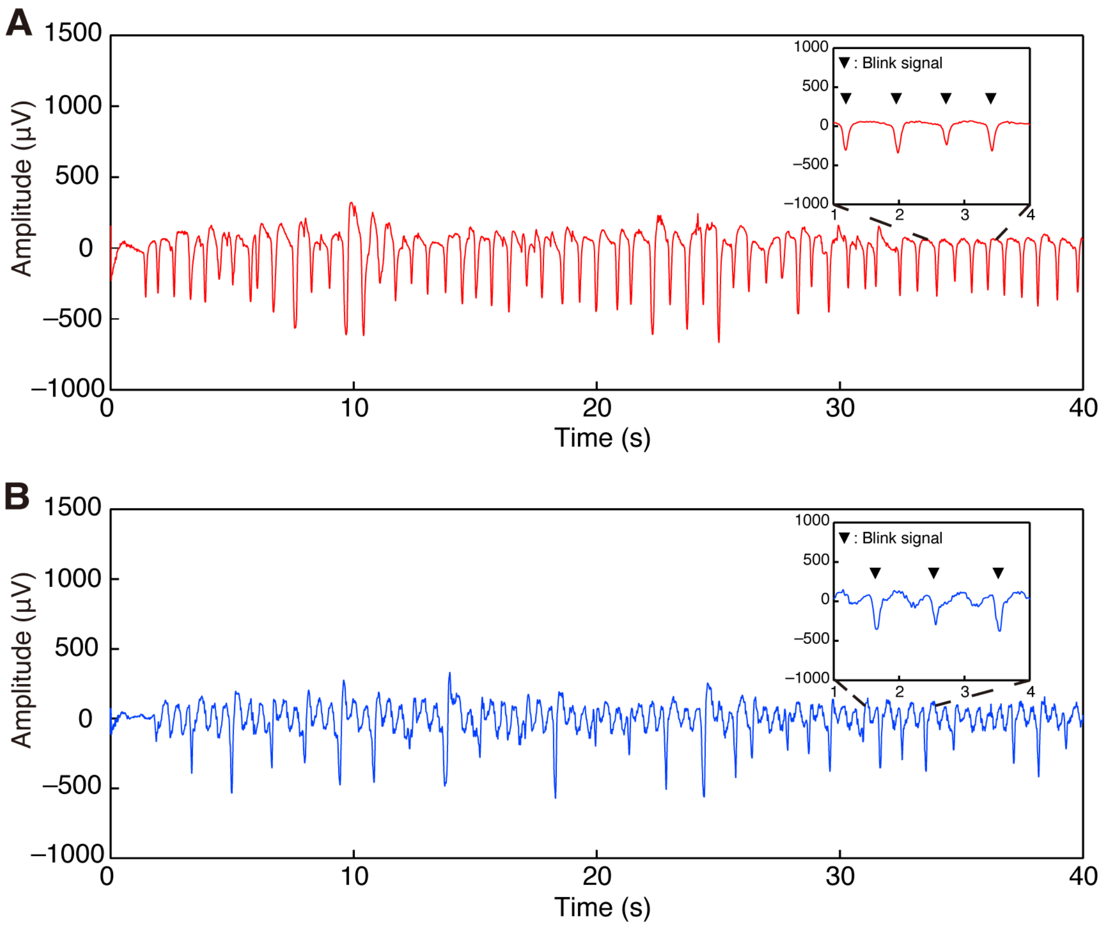

Fig. S32. EOG signals recorded during dynamic motion. (A) EOG signals were measured while the wearer was walking. **(B)** EOG signals were measured while the wearer was jumping. In both conditions, the wearer performed blinks to generate clear EOG events, as shown in the insets.

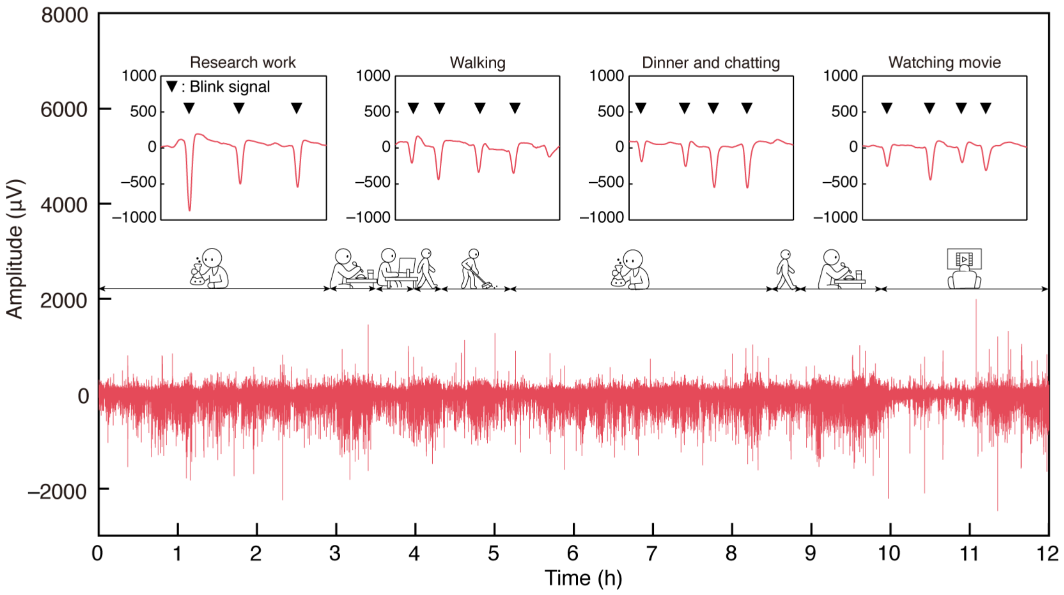

Fig. S33. Long-term EOG monitoring over 12 hours of daily life activities, including research work, walking, dinner and chatting, and watching movies. The insets show part of the blinking waveforms (marked by arrowheads) recorded at different activities.

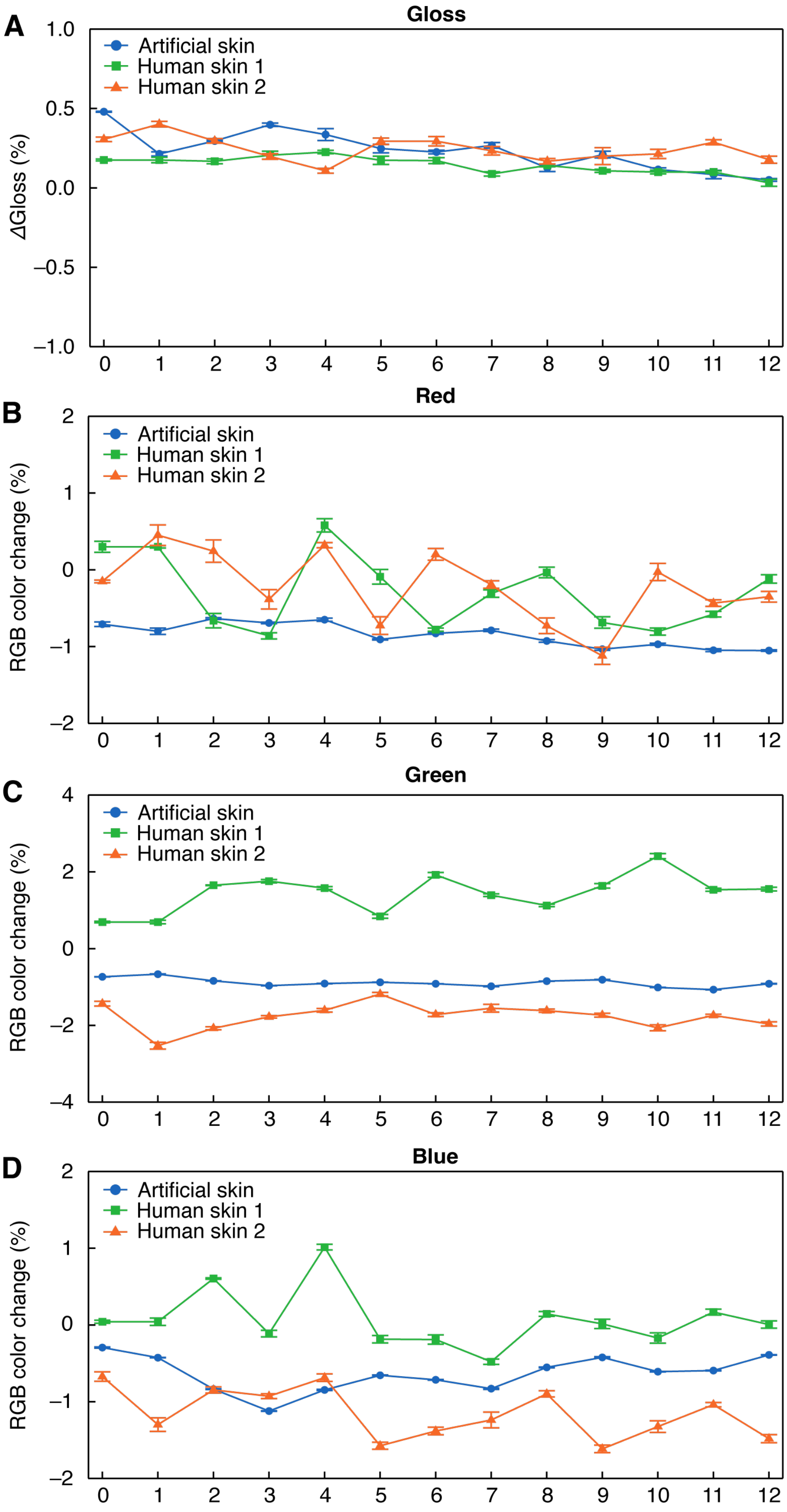
A
Gloss
Artificial skin
Human skin 1
Human skin 2
ΔGloss (%)
1.0
0.5
0.0
−0.5
−1.0
0 1 2 3 4 5 6 7 8 9 10 11 12
B
Red
RGB color change (%)
2
1
0
−1
−2
C
Green
4
2
0
−2
−4
D
Blue

Fig. S34. Time-dependent changes in surface gloss and RGB color of the invisible electrode on skin over 12 h. Gloss **(A)** and RGB values **(B, C, D)** were recorded after transfer onto artificial skin, Volunteer 1 (female), and Volunteer 2 (male). Measurements were conducted at 1-hour intervals to assess the stability of optical properties during long-term wear. The artificial skin exhibiting stable color over time, was used as the primary reference for color analysis. Error bars denote standard error.

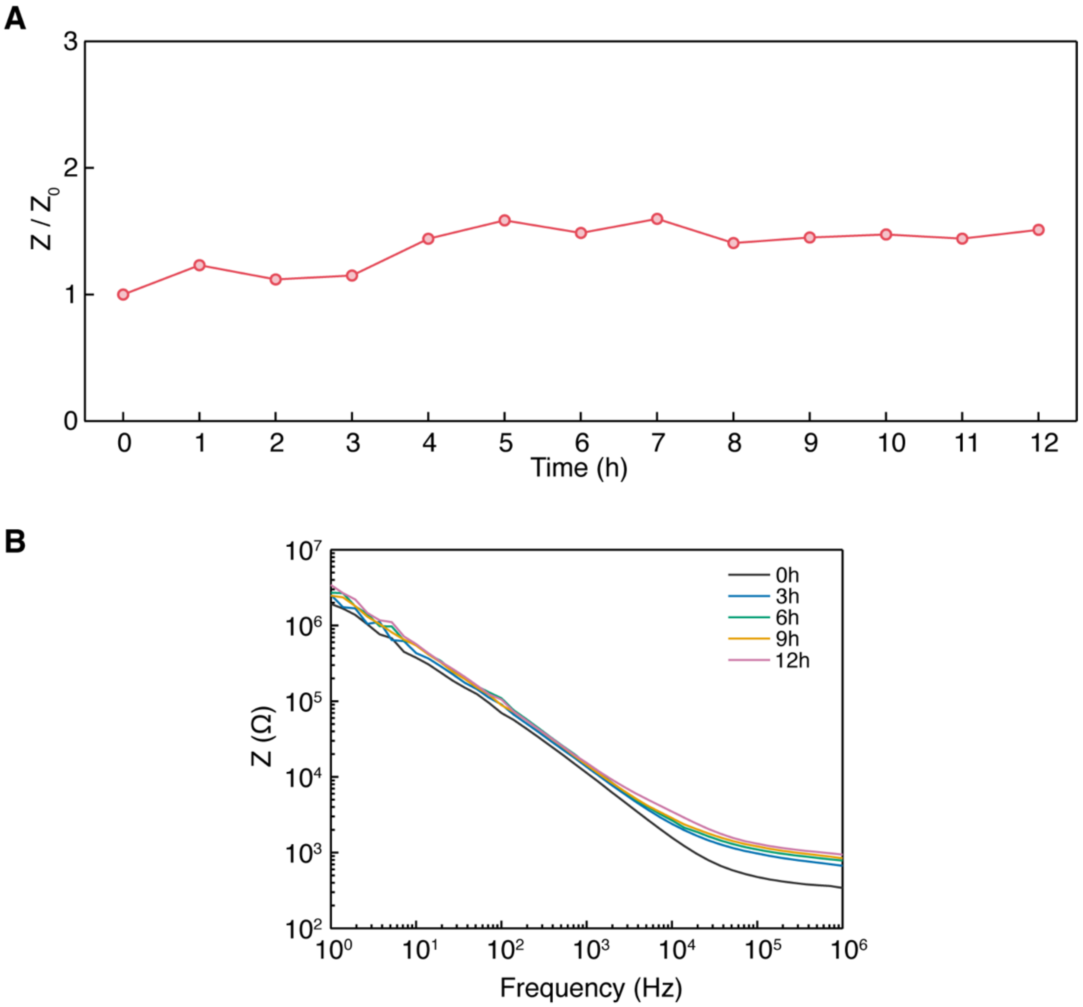

Fig. S35. Long-term stability of the skin impedance of the invisible electrodes. (A) Time-dependent change in skin impedance measured at 10 Hz during 12 h continuous wear. **(B)** Frequency-dependent skin impedance spectra measured at 0, 3, 6, 9, and 12 h.

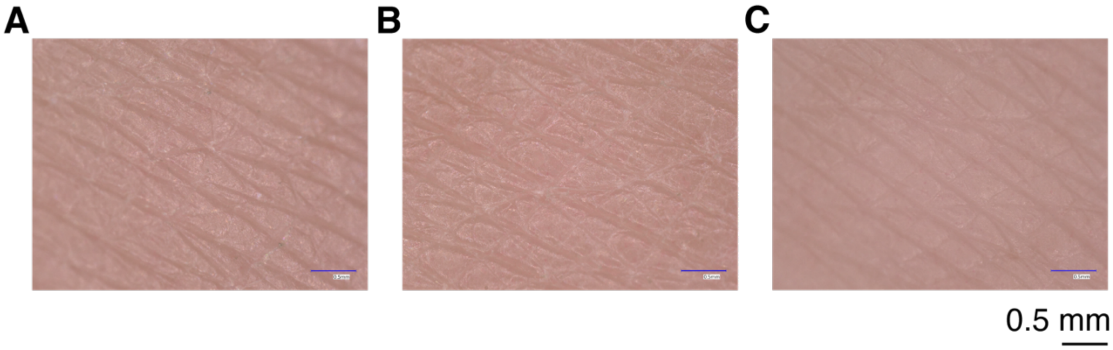

Fig. S36. Skin compatibility of the invisible electrode after long-term wear. Optical microscope images of the skin surface **(A)** right after attaching the film on skin, **(B)** after 12 h of wear, and **(C)** after film removal at 12 h. No visible irritation, such as redness or inflammation, is observed after long-term use, indicating good skin compatibility. Scale bars, 0.5 mm.

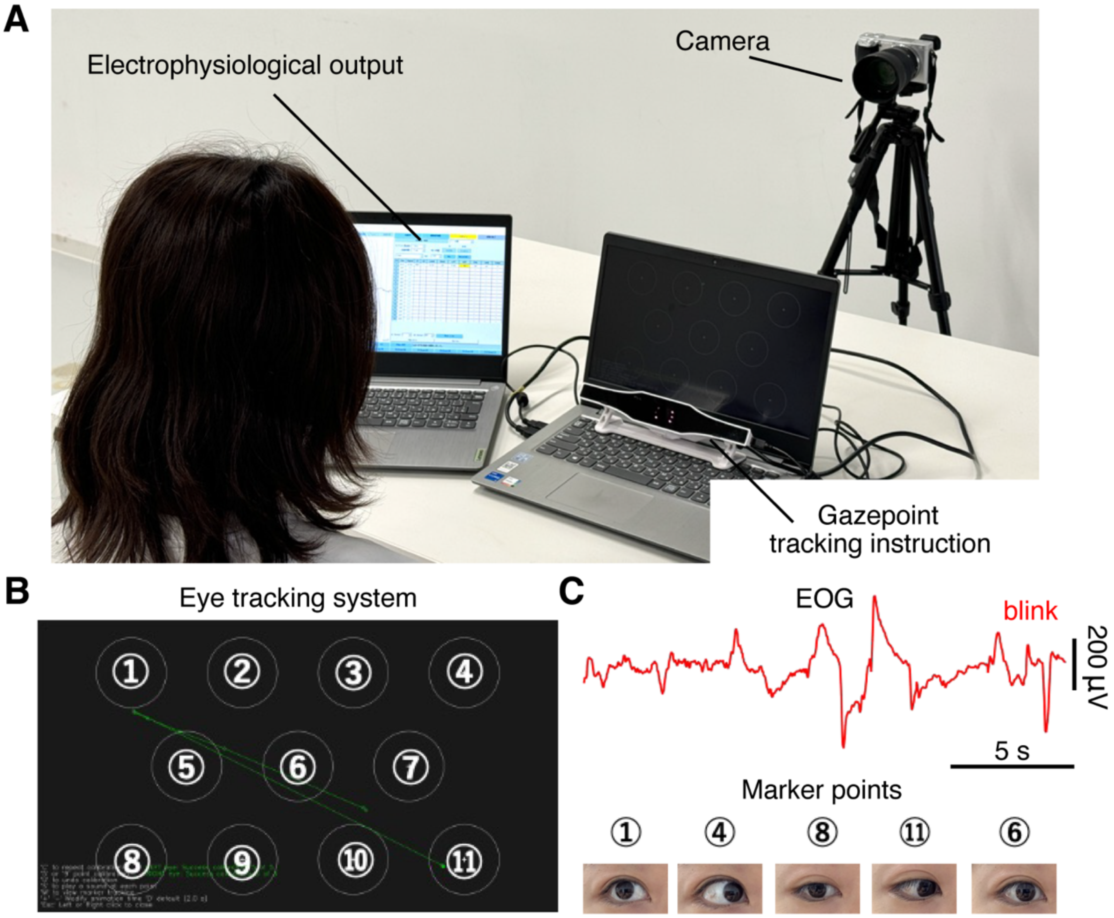

Fig. S37. An EOG measurement setup. (A) A photo of the entire system. Volunteer utilized the invisible electrode to measure the EOG signal while the camera and videooculography validated the eyes' position. **(B)** An image shown on the display during videooculography. **(C)** Result of simultaneous measurement of eyes using EOG by invisible electrodes, a camera, and a videooculography. The numbers in the middle are marker points obtained from the videooculography. The lowers are the direction the eyes obtained from the camera.

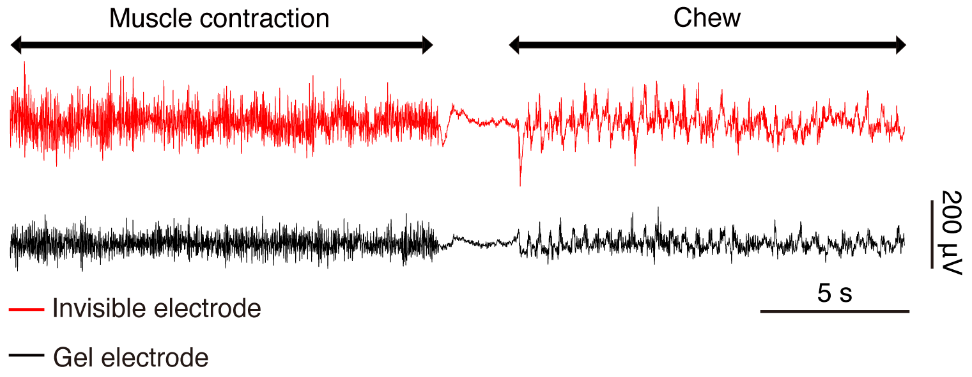

Fig. S38. Facial EMG responses to muscle contraction and chewing were recorded using both the invisible and gel electrodes.

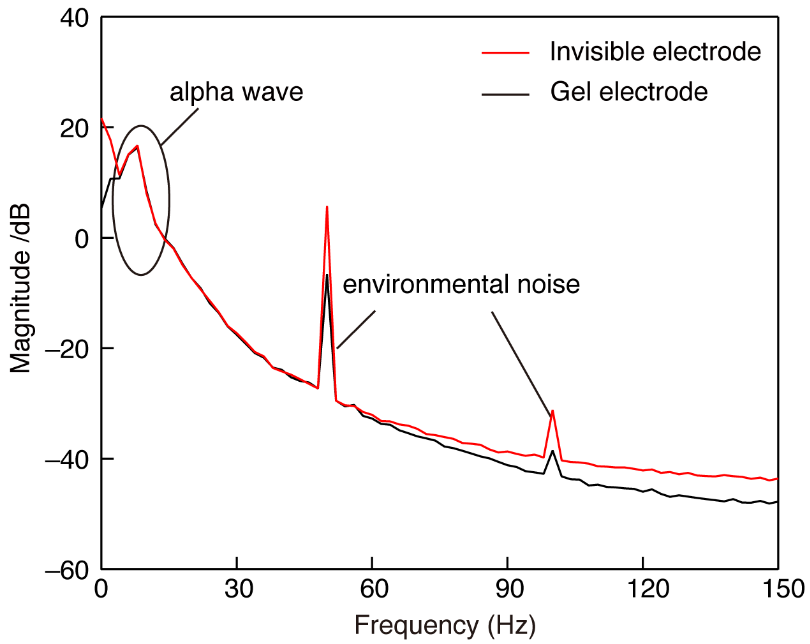

Fig. S39. PSD of EEG signals acquired using the invisible electrode (red) and the gel electrode (black). The invisible electrode exhibits higher spectral magnitude in the low-frequency band (8-13 Hz), where alpha-wave activity was observed.

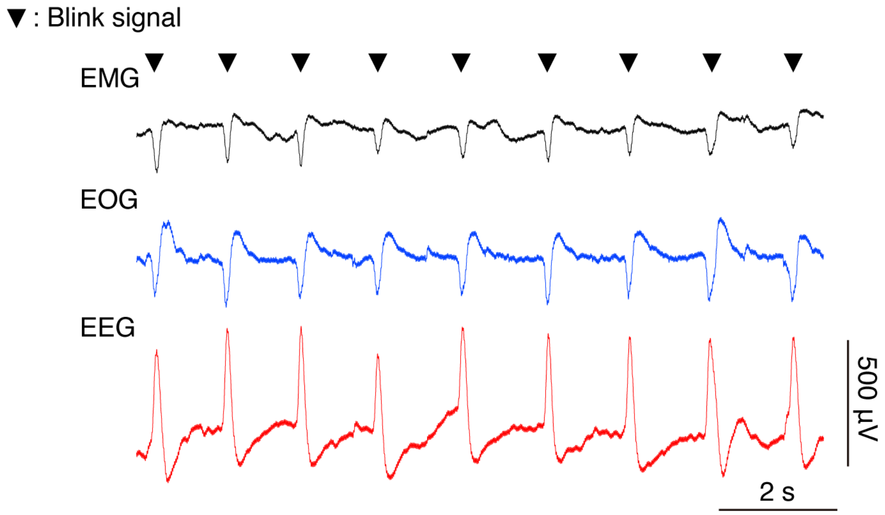

Fig. S40. Simultaneous measurements of EMG, EOG, and EEG signals during blinking events, using the invisible electrode. Invisible electrodes were attached to the cheeks to measure EMG signals, to the area below the eyelids to measure EOG signals, and to the forehead to measure EEG signals. Blink events (▼) show synchronized responses across all three channels. All three signals showed clear signals to blinking.

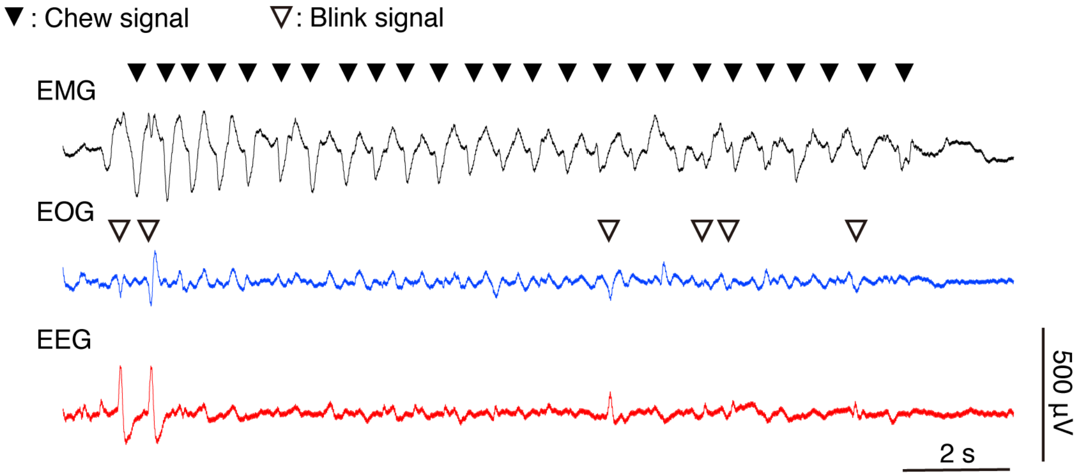

Fig. S41. Simultaneous record of chew signals from EMG, EOG, and EEG using the invisible electrode. Invisible electrodes were attached to the cheeks to measure EMG signals, to the area below the eyelids to measure EOG signals, and to the forehead to measure EEG signals. The EMG responses to chew signals are the most noticeable. Chew events (▼) and blink events (▽) show synchronized responses across all three channels.

Supplementary Table 1. Comparison of various skin-like electrodes

	Conductivity	Stretchability	Transparency	Visual invisibility	Tactile invisibility	Thickness	Young's modulus	Breath ability	Function
Skin-colored films for concealing on-skin electronic devices[58]	×	△	×	Skin-simulate	N/A	N/A	N/A	△	Makeup for facial expressions and interactive experiences.
Mesh-like nano-gold electrode for human motion detection[59]	◎ (1Ω Sq.$^{-1}$)	○ (~80%)	×	Gold color	N/A	430 nm	N/A	◎	Motion activity monitoring.
Transparent electronic skin with ultrathin elastic gold nanofibers[60]	◎ (7Ω Sq.$^{-1}$)	○ (~75%)	◎ (86%)	High gloss	N/A	10 μm	1.3 MPa	○	Electronic skin features self-powered sensing.
Multimodal sensing MXene electronic skin[61]	○ (11000 S/cm)	N/A	◎ (89%)	Dark gloss	N/A	20 nm	N/A	◎	Sensing of neuroelectric signals.
Biomimetic skin-like super tattoo[62]	◎ (16 mS·m^{-1})	N/A	○ (78%)	High gloss	N/A	2 μm	>0.5 MPa	◎	Monitoring weak EOG of swallowing.
Imperceptible electrooculography graphene sensor system[55]	N/A	○ (~50%)	◎ (85%)	Dark color	N/A	350 nm	N/A	◎	Monitoring of EOG signals for human-robot interface.
Stretchable transparent electrodes[42]	◎ (20 Ω Sq.$^{-1}$)	○ (~100%)	◎ (88%)	High gloss	N/A	30 μm	6 MPa	△	Wearable OPDs for heart rate detection.
Self-powered, ultrathin, and transparent printed pressure sensor[63]	△1000 S/cm	N/A	△ (~58%)	High gloss	N/A	7 μm	N/A	○	Bio signal monitoring.
Ultra-thin, transparent, anti-freezing organohydrogen[64]	N/A	◎ (~323%)	◎ (98%)	High gloss	N/A	0.1 mm	N/A	△	Detecting changes in humidity, temperature, and mechanical strain.
Untraconformal transparent electronic skin[38]	◎ (33 Ω Sq.$^{-1}$)	△ (~8%)	◎ (86%)	Visible micro-circuits	N/A	160 nm	4.1 GPa	◎	Monitoring of EMG and ECG signals.
Invisible electrode (this work)	○ (~170 Ω Sq.$^{-1}$)	○ (>;'70%)	◎ (>85%)	Invisible	Same as skin	200 nm	9.78 MPa	◎	Monitoring of facial electrophysiological signals.

Movie captions:

Movie S1. The process for transferring the invisible electrode onto the hand.

Movie S2. Real-time monitoring of the EOG signals by the invisible electrode.

Movie S3. Continuous EOG signals were obtained while the wearer was walking and jumping.
